# Substrate-driven shifts in microbial communities reshape metabolite profiles during soybean residue fermentation

**DOI:** 10.3389/fmicb.2026.1919307

**Published:** 2026-09-28

**Authors:** Gaorui Bian, Yamei Gu, Yaoxing Tang, Kaidong Deng, Qian Tang, Minyang Zhang, Weiyun Zhu, Yuyang Yin

**Affiliations:** 1 Department of Animal Nutrition and Feed Science, College of Animal Science and Food Engineering, Jinling Institute of Technology, Nanjing, China; 2 Laboratory of Gastrointestinal Microbiology, College of Animal Science and Technology, Nanjing Agricultural University, Nanjing, China; 3 Huzhou Academy of Agricultural Sciences, Huzhou, China

**Keywords:** fermentation duration, fermentation temperature, metabolite profile, microbial community, soybean residue fermentation, substrate supplementation

## Abstract

Fermentation is an effective strategy to prolong the shelf life of soybean residue and improve its value as an animal feed resource. To investigate the influence of differing fermentation conditions on the microbial community and metabolite profile of fermented soybean residue, fresh soybean residue was fermented in sealed anaerobic bag using a mixed inoculum comprising *Bacillus subtili*s, *Lacticaseibacillus rhamnosus*, *Limosilactobacillus reuteri*, and *Lactiplantibacillus plantarum*, with varying levels of wheat bran supplementation (0–15%), fermentation temperatures (24–37 °C), and durations (24–72 h). The microbial community and metabolite profiles were analyzed after fermentation. The results indicated that wheat bran supplementation was the primary factor driving microbial community shift (explaining 49.1% of the axes contributions, *P* = 0.002), inducing a transition from Proteobacteria-dominated to Firmicutes-dominated communities. The shift was characterized by targeted enrichment of *Bacillus* and multiple genera of lactic acid bacteria, alongside suppression of potential pathogens, such as Enterobacteriaceae and *Pseudomonas*. Concurrently, the process of fermentation resulted in a substantial increase in the levels of functional metabolites, including tryptophan, indolelactic acid, and various phenolic acids, accompanied by a reduction in flavonoid content. Fermentation temperature was the primary factor influencing metabolite distribution, explaining 18.1% of the axes contributions (*P* = 0.002). Network analysis revealed close correlations between specific microbiota (e.g., *Lactobacillus*, *Limosilactobacillus*) and key metabolites, including aromatic lactic acid metabolites. The findings presented herein provide insights into substrate-driven microbial modulation during soybean residue fermentation, thus establishing a foundation for optimizing fermentation conditions to enhance feed quality for animal health applications.

## 1 Introduction

Soybean residue is a protein- and carbohydrate-rich byproduct of soybean processing with potential as an animal feed (Rahman et al., 2021; Vong and Liu, 2016). However, its high moisture content causes rapid deterioration and poses storage challenges (Vong and Liu, 2016). To address these limitations, solid-state fermentation has been applied to extend its preservation and improve its nutritional availability for feed applications (Jiang et al., 2019).

Fermentation of feed materials is the result of the interaction between inoculated strains and the indigenous microbiota under environmental conditions (Yan et al., 2025). *Bacillus subtilis* is widely used in solid-state fermentation of soybean and agro-industrial residues for its proteolytic and growth-promoting properties (Arpit Singh et al., 2022; Huang et al., 2023). *Lacticaseibacillus rhamnosus*, *Limosilactobacillus reuteri*, and *Lactiplantibacillus plantarum* are standard probiotic lactic acid bacteria (LAB) inoculants for silage and feed fermentation, known for rapid acidification and suppression of spoilage organisms (Carvalho et al., 2021; Sharma et al., 2023). Fermentation performance is strongly modulated by environmental factors such as temperature and nutrient availability (Kampen, 2014; Li et al., 2019). Co-fermented materials supply carbon, nitrogen and other essential nutrients required for microbial growth, and different microbial taxa exhibit distinct substrate selectivity (Konopka et al., 1998). Accordingly, nutrient substrates and supplementary ingredients (such as wheat bran) can selectively filter specific microbial community structures and determine overall fermentation patterns (Chatellard et al., 2016). Fermentation temperature exerts a critical influence on microbial enzyme activity and catalytic efficiency (Lee et al., 2014; Wang et al., 2016). As the fermentation process continues, shifts in microbial composition and diversity, driven by fermentation conditions, also exert a significant influence on the composition of metabolic products (Chen et al., 2025). However, systematic multi-factor research exploring the combined effects of fermentation parameters on the microbiota and metabolome of soybean residue remains limited.

Fermentation of plant-based substrates promotes microbial biotransformation, giving rise to multiple classes of bioactive metabolites. Carbohydrate fermentation leads to the production of short-chain fatty acids, including lactic, acetic, and propionic acid (Gänzle, 2014). Meanwhile, aromatic amino acids such as tryptophan and phenylalanine are converted into indolelactic acid (ILA) and phenyllactic acid (PLA) via transamination (Dodd et al., 2017; Plenge et al., 2026). Additionally, microbial esterase and decarboxylase activities release phenolic acids—such as *trans*-cinnamic acid, 5-methoxysalicylic acid, and homoveratric acid—from their bound forms (Kumar et al., 2025; Liu et al., 2026).

Raw soybean residue is particularly noteworthy as a fermentation substrate, as it provides not only proteins and carbohydrates but also a rich pool of flavonoids and polyphenols, including isoflavones, genistein, and daidzin (Carneiro et al., 2020). During fermentation, endogenous bacterial enzymes catalyze the biotransformation of these phytochemicals (Das and Rosazza, 2006). For example, isoflavone glycosides—daidzin, genistin, and glycitin—are hydrolyzed by β-glucosidase to their aglycone forms (daidzein, genistein, and glycitein), which are more bioavailable and biologically potent (Hati et al., 2015). The efficiency and specificity of these conversions are governed by fermentation parameters (Handa et al., 2014), and the resulting metabolites have implications for animal health and nutrition.

Although individual studies have examined the effects of single fermentation parameters on soybean residue quality (Jiang et al., 2019; Lian et al., 2019), systematic multi-factor investigations exploring the combined and relative contributions of substrate supplementation, temperature, and duration on both the microbiota and metabolome of fermented soybean residue remain limited. Furthermore, while the health-promoting effects of specific metabolites (e.g., indolelactic acid) have been demonstrated in mammalian models (Laursen et al., 2021; Wang et al., 2025), the coordinated microbial-metabolite relationships during substrate-driven fermentation of soybean residue have not been systematically characterized.

To investigate the shifts in the microbial community and metabolite profiles of soybean residue under different fermentation conditions, this study aimed to: (1) establish a comprehensive factorial experimental design that enabled quantification of the independent and relative explanatory power of three key fermentation parameters on community and metabolite dynamics; (2) integrated multi-omics analysis (16S rRNA sequencing + untargeted metabolomics) to establish microbial-metabolite correlation networks; and (3) to identify substrate-driven targeted enrichment of probiotic genera and associated functional metabolites with documented immunomodulatory and gut barrier-enhancing activities. Centered on livestock health promotion, this work intends to screen optimal fermentation regimes, advance the comprehensive utilization of soybean processing byproducts, and support sustainable development of the animal husbandry industry.

## 2 Materials and methods

### 2.1 Experimental design

Fresh soybean residue samples were collected from Huzhou City, Zhejiang Province, China. In order to simulate industrial solid-state fermentation conditions for feed production, the soybean residue was used in its raw, unsterilized form, and the wheat bran used was dry and stored at room temperature. It should be noted that raw materials are typically not sterilized due to practical and economic considerations. While the native microbiota present in the raw material could potentially contribute to fermentation, the inoculated strains were intended to establish dominance and drive the fermentation process, consistent with industrial practice. The fermentation inoculum was composed of four bacterial strains: *Bacillus subtilis*, *Lacticaseibacillus rhamnosus*, *Limosilactobacillus reuteri*, and *Lactiplantibacillus plantarum*, which were obtained from the culture collection of our laboratory. *Bacillus subtilis* was isolated from fermented soybean meal, while *Lacticaseibacillus rhamnosus* (CCTCC M20232713), *Limosilactobacillus reuteri* (CCTCC M20232531), and *Lactiplantibacillus plantarum* (CCTCC M20251743) were isolated from traditional fermented food and stored in China center for type culture collection (CCTCC). Each strain was cultured in de Man, Rogosa and Sharpe (MRS) broth at 37°C for 24 h to reach a cell density of > 108 CFU/mL. The four strains were mixed in a 1:1:1:1 (v/v) ratio to prepare the composite inoculum.

The soybean residue samples were randomly divided into 8 groups and 1 control group (Table 1). For each fermented treatment, 100 g of co-fermented material (soybean residue and wheat bran) was weighed into a vacuum-sealed anaerobic bag and inoculated with 5% (v/w) composite bacterial suspension. Four biological replicates were set for every group, and all bags were vacuum sealed for static anaerobic fermentation. Detailed experimental grouping is displayed in Table 1. Group 0 served as the unfermented raw soybean residue control. Temperature optimization groups (1–3) contained no wheat bran, maintained a fixed fermentation duration of 48 h, and were incubated at 24, 28, and 37 °C, respectively. Substrate optimization groups (4–6) were fermented at 37 °C for 48 h with wheat bran supplementation rates of 5, 10, and 15%, respectively. Duration optimization groups (7, 6, and 8) received 15% wheat bran and were incubated at 37 °C for 24, 48, and 72 h, respectively.

**TABLE 1:** The experimental design for soybean residue fermentation in present study.^1^

Group	Co-fermented materials (g)	Soybean residue (%)	Wheat bran (%)	Fermentation durations (h)	Fermentation temperature (°C)	Crude protein (%)^2^	Water soluble carbohydrates (%)
0	100	100	/	/	/	4.9	3.9
1	100	100	0	48	24	4.9	3.9
2	100	100	0	48	28	4.9	3.9
3	100	100	0	48	37	4.9	3.9
4	100	95	5	48	37	5.4	4.0
5	100	90	10	48	37	6.0	4.1
6	100	85	15	48	37	6.5	4.2
7	100	85	15	24	37	6.5	4.2
8	100	85	15	72	37	6.5	4.2

^1^Group 0 represented untreated raw soybean residue without inoculation or fermentation. Groups 1–3 were fermented without wheat bran at three temperature gradients for 48 h. Groups 4–6 received graded wheat bran additions (5, 10, 15%) and fermented at 37 °C for 48 h. Groups 7 and 8 contained 15% wheat bran and fermented at 37 °C for 24 and 72 h, respectively. Group 6 (15% wheat bran, 37 °C, 48 h) represents both the optimal substrate level identified from Groups 4–6 and the intermediate time point for the duration gradient (Groups 7–8). All fermented groups (1–8) received a uniform 5% (v/w) mixed bacterial inoculant.

^2^Crude protein and water-soluble carbohydrate values were calculated based on formulation; batch-specific composition was not determined.

At each designated fermentation endpoint (24, 48, or 72 h), the sealed bag was opened once, and two subsamples were immediately collected for subsequent analyses. For pH measurement, one subsample was homogenized with purified water at a 1:1 ratio (w/v), and the pH value was determined immediately using a calibrated pH meter. The pH of the fermentation system was detected at each experimental endpoint but was not externally adjusted during the fermentation process. Biological replicates were independent bags; no bag was re-sampled. This approach was adopted to reflect the natural pH dynamics driven by microbial metabolism, consistent with typical solid-state fermentation practices in feed production. The pH values of all fermentation groups were significantly lower than those of the non-fermented group, indicating that the fermentation of soybean residue under different conditions achieved the expected results (Supplementary Figure 1).

### 2.2 Microbial sequencing of fermented soybean residue

Samples were numbered according to the experimental design groups and sequenced using paired-end sequencing on the Illumina MiSeq platform. Genomic DNA was extracted from the samples using the QIAamp^®^ DNA Stool Mini Kit (QIAGEN, Germany), followed by assessment of DNA concentration and purity. DNA concentration was quantified using Pico Green fluorescence, and the products were purified and recovered using the QIAGEN QIAquick PCR Purification Kit. Bacterial 16S rRNA gene sequences were amplified by forward primer 338F (5’-ACT CCT ACG GGA GGC AGC A-3’) and reverse primer 806R (5’-GGA CTA CHV GGG TWT CTA AT-3’), and the amplified products underwent Pico Green fluorescence quantification and Agilent 2200 TapeStation electrophoresis. After quality control, the samples were sequenced on the MiSeq (Rapin et al., 2017).

Raw sequencing data were processed using QIIME2 (version 2021.8) (Bolyen et al., 2019). Quality filtering, denoising, and chimera removal were performed using the DADA2 pipeline (Callahan et al., 2016), and Amplicon sequence variants (ASVs) were filtered using a threshold of 0.005% of the total number of sequenced sequences. Taxonomic assignment was performed using the SILVA 138 reference database (Quast et al., 2013). Principal coordinates analysis (PCoA) was conducted using Bray-Curtis distance.

### 2.3 High-performance liquid chromatography-mass spectrometry (HPLC-MS) analysis of fermented soybean residue

The samples for HPLC-MS analysis were the same as those for microbial analysis. 20 mg of fermentation sample was mixed with 200 μL of methanol, vortexed for 30 s, incubated at -20 °C for 1 h, and centrifuged at 15,000 g and 4 °C for 15 min. The supernatant (100 μL) was dried and reconstituted in 100 μL LC–MS grade methanol.

LC separation was performed on an Atlantis T3 column (100 × 2.1 mm, 3.0 μm) with a gradient of 5 mM ammonium formate with 0.05% formic acid (A) and acetonitrile (B) at 0.25 mL/min (injection 5 μL): 0–3 min at 5% B, 3–8 min 5–65% B, 8–10 min 65–95% B, 10–12.5 min 95% B, 12.5–13 min 95–5% B, and 13–17 min 5% B. For polar compounds, an XBridge BEH Amide column (4.6 × 100 mm, 3.5 μm) was used with 15 mM ammonium acetate with 0.3% ammonia (pH 9) (A) and acetonitrile (B) at 0.4 mL/min (injection 5 μL): 0–1 min at 85% B, 1–12 min 85–30% B, 12–13 min 30% B, 13–14 min 30–85% B, and 14–27 min 85% B. The 6545 XTQ-TOF mass spectrometer was operated with a spray voltage of -3.5 kV in negative ion mode and +4 kV in positive ion mode. The drying gas flow rate was set at 9 L/min, and the sheath gas flow rate was set at 110 L/min, with a sheath gas temperature of 325 °C. Fast data-dependent acquisition (DDA) MS/MS experiments were performed with a collision energy ramp over the mass range of 50–1,000 m/z.

To ensure quality control and assurance for LC-MS/MS analysis, pooled quality control (QC) samples were prepared by mixing equal aliquots of all sample extracts and injected every 10 samples throughout the analytical sequence to monitor instrument performance and signal stability. The coefficient of variation (CV) for retention time was < 1.5% across all QC injections, and CV for peak area was < 20% for > 85% of detected metabolites. Blank samples (methanol) and calibration standards were analyzed in parallel. The LC separation protocol followed standardized metabolomics guidelines as recommended by the Metabolomics Standards Initiative (MSI) (Sumner et al., 2007). All solvents used were LC-MS grade, and mobile phase buffers were freshly prepared daily with ultrapure water (18.2 MΩ⋅cm).

Peak picking and alignment were performed using Progenesis QI software (Nonlinear Dynamics, Newcastle, United Kingdom). Molecular identification of the detected metabolites was achieved by matching the acquired precursor and fragment ion spectra against several standard metabolome databases, including the Human Metabolome Database (HMDB),^1^ MassBank,^2^ and METLIN.^3^ Partial identification was further confirmed by comparison with authentic standards. Data normalization and statistical analysis were performed using the web-based platform MetaboAnalyst 6.0.^4^ Peak area data were normalized by sample median, log-transformed, and autoscaled.

### 2.4 Data analysis

Raw data were organized using Microsoft Excel 2021. One-way analysis of variance (ANOVA) was performed on the detected data using SPSS (Version 26.0), Tukey’s test was applied for *post-hoc* comparisons, and differences with FDR-adjusted *P* < 0.05 were considered statistically significant. All results were expressed as “mean ± standard error.” Redundancy analysis (RDA) of microbial and metabolite relative abundances was performed using Canoco 5 software, using Partial Monte Carlo Permutation Test to calculate the contribution of each factor to the explanatory power of the microbiota or metabolites (Ramette, 2007). A co-occurrence network between the relative abundances of microbiota and metabolites was conducted using Debiased Sparse Partial Correlation (DSPC) method (Basu et al., 2017).

## 3 Results

### 3.1 Effects of different fermentation conditions on microbial distribution of fermented soybean residue

A total of 2,097,266 clean reads were sequenced in all samples. Rarefaction curves for all samples gradually plateaued with increasing sequencing depth (Figure 1A), indicating that sequencing coverage sufficiently captured the full microbial diversity within each sample.

**FIGURE 1:**
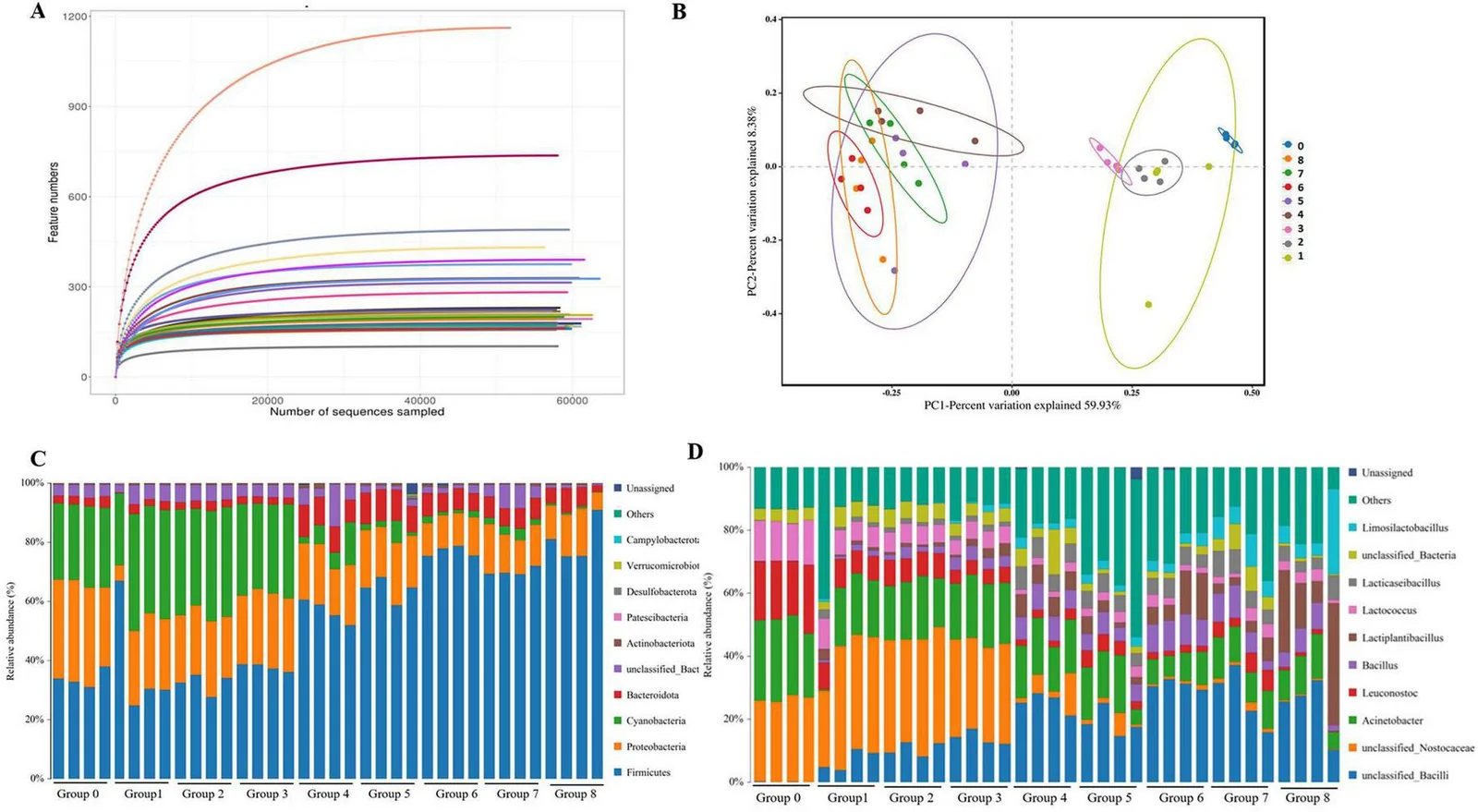
Microbial community distribution across all soybean residue samples. **(A)** Rarefaction curves of microbiota in fermented soybean residue for all samples. **(B)** PCoA plots of the microbial community of fermented soybean residue for all samples based on the Bray–Curtis method. **(C)** Relative abundance of microbial community of fermented soybean residue at the phylum level for all samples. **(D)** Relative abundance of microbial community of fermented soybean residue at the genus level for all samples.

Bray-Curtis PCoA plot (Figure 1B) revealed clear separation across treatment groups. Samples from different groups were divided into three distinct clusters: unfermented samples (Group 0) and groups without supplemental wheat bran (Groups 1–3), and groups with supplemental wheat bran (Groups 4–8). The three clusters were arranged sequentially along the X-axis, showing a progressive shift in microbial community structure from unfermented samples to those fermented with or without additional substrates. Samples from Groups 1, 5, and 8 exhibited high intra-group dispersion, suggesting higher inter-replicate variability within these treatments. The tight clustering and low biological replicate variability were observed in Groups 0, 2, 3, 4, 6, and 7, indicating that intra-group variation in microbial composition was minimal among these groups. Notably, the distribution area of Group 1 partially overlapped with those of Group 0 and Group 2.

As shown in Figure 1C, the microbial communities of all samples at the phylum level were primarily composed of phyla Firmicutes, Proteobacteria, Cyanobacteria, Bacteroidota, unclassified Bacteria, and Actinobacteriota. These phyla accounted for 99% of the microbial abundance across all samples. Proteobacteria was found to be dominant in the unfermented samples (Group 0) and the groups without added wheat bran (Groups 1–3), whereas *Firmicutes* became the dominant phylum in all wheat bran-amended treatments.

The top 10 dominant genera are shown in Figure 1D. The microbial communities in all samples were primarily composed of unclassified Bacilli, unclassified Nostocaceae, *Acinetobacter*, *Leuconostoc*, *Bacillus*, *Lactiplantibacillus*, *Lactococcus*, *Lacticaseibacillus*, unclassified Bacteria, *Limosilactobacillus*, and other genera.

### 3.2 Consistent directional shifts in relative abundances of bacterial genera during fermentation

As shown in Figure 2A, comparative analysis across treatment groups with progressively intensified fermentation parameters (increasing wheat bran supplementation, fermentation temperature, and duration) revealed that some genera exhibited a consistent decreasing abundance trend from Group 1 to Group 8 in fermented soybean residue (FDR-adjusted *P* < 0.05). The relative abundances of genera *Leuconostoc*, *unclassified Nostocaceae, Lactococcus*, and *Acinetobacter* were found to reach their highest values in Groups 2 and 3. The relative abundances of unclassified Enterobacteriaceae, *Enterobacter*, *Pseudobacillus*, and *Pseudomonas* in fermented soybean residue were significantly lower than those in the raw soybean residue (FDR-adjusted *P* < 0.05).

**FIGURE 2:**
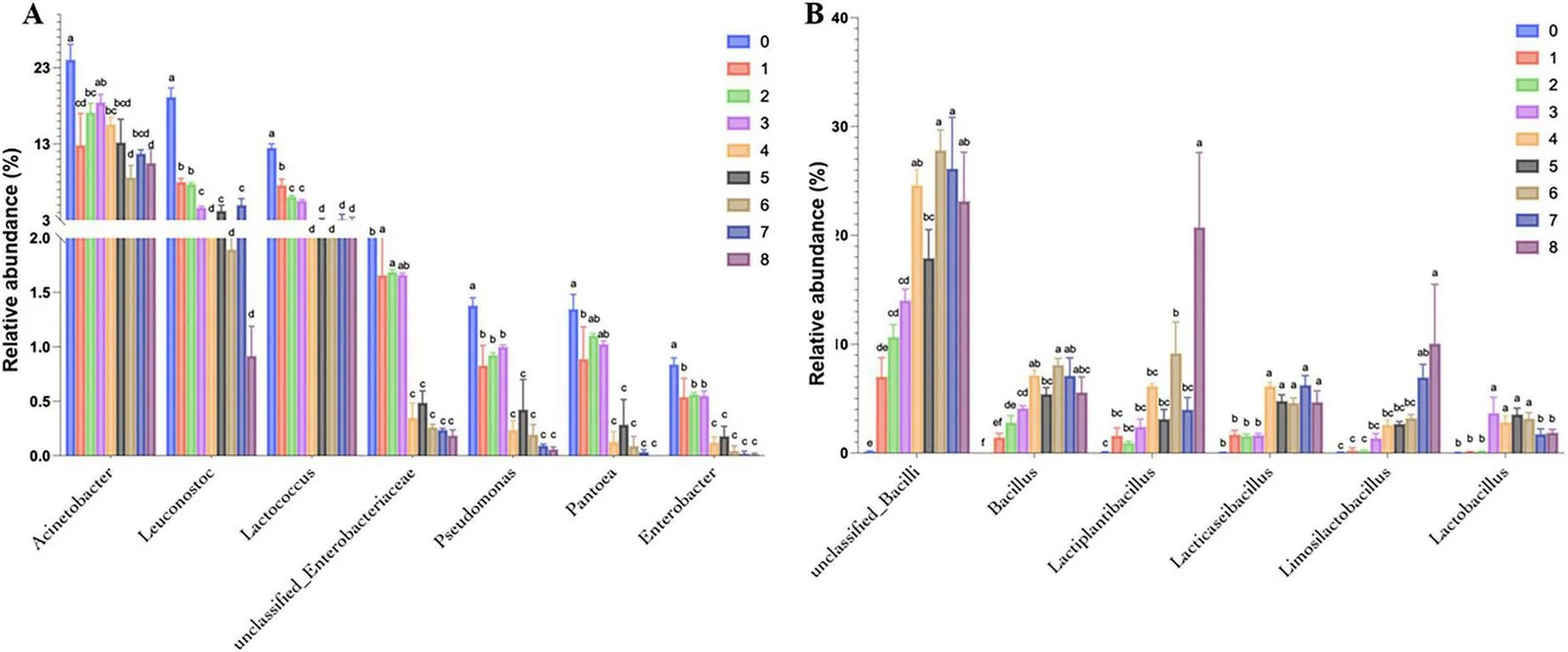
Bacterial genera with significantly altered relative abundances across fermentation treatments. **(A)** The genera with relative abundance decreased in proportion to the increase in co-fermented material, fermentation temperature and fermentation duration (FDR-adjusted *P* < 0.05). **(B)** The genera with relative abundance increased in proportion to the increase in co-fermented material, fermentation temperature and fermentation duration (FDR-adjusted *P* < 0.05). Different lowercase letters indicate significant differences among groups (FDR-adjusted *P* < 0.05).

Conversely, the relative abundances of genera *Bacillus*, *Lactiplantibacillus*, *Lacticaseibacillus*, *Limosilactobacillus*, and *Lactobacillus* exhibited a consistent upward trend across Groups 1–8 (FDR-adjusted *P* < 0.05) as shown in Figure 2B. Their relative abundances were highest in wheat bran-supplemented groups (4–8), intermediate in non-supplemented fermented groups (1–3), and extremely low in the unfermented Group 0.

### 3.3 Effects of different fermentation conditions on metabolites profiles of fermented soybean residue

A total of 239 metabolites were detected among all samples (Supplementary Table S1). The PCA of all detected metabolites displayed widespread dispersion across treatment groups (Figure 3A). The unfermented soybean residue was completely separated from all fermented treatments (Groups 1–8), while the groups without substrate addition (Groups 1–3) were close to each other, and the groups with substrate addition (Groups 4–8) exhibited greater inter-group separation, verifying that fermentation drastically reshaped the metabolite composition of soybean residue.

**FIGURE 3:**
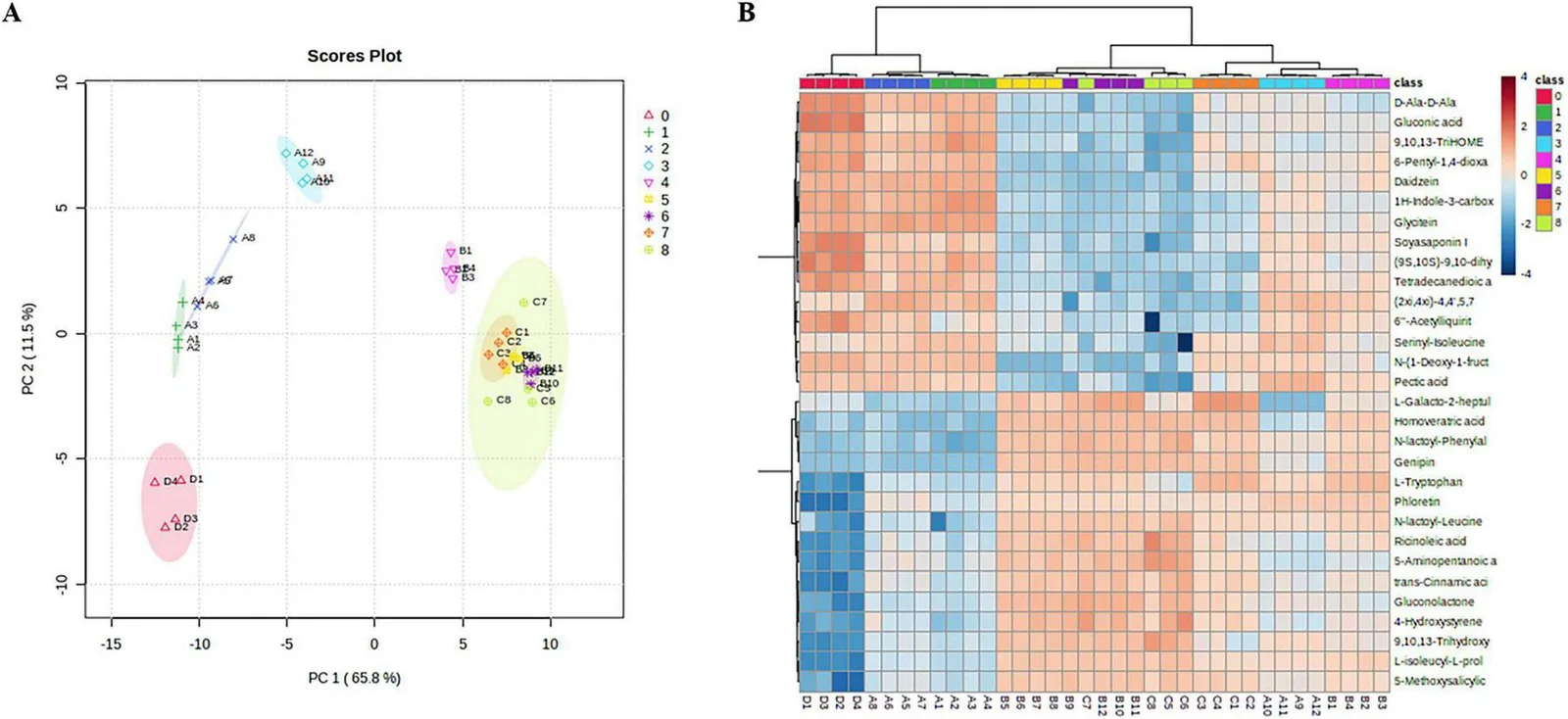
The metabolites distribution among all samples. **(A)** The principal component analysis plot of the distribution of metabolites across all samples. **(B)** The heatmap of the distribution of metabolites across all samples.

As shown in Figure 3B, the samples were broadly divided into three clusters: Cluster 1 (Groups 0, 1, and 2), Cluster 2 (Groups 3, 7, and 4), and Cluster 3 (Groups 5, 6, and 8). Cluster 1 contained high levels of Daidzein, Soyasaponin I, Glycitein, Gluconic acid, Pectic acid, 9,10,13-triHOME, and 6-pentyl-1,4-dioxan-2-one. Cluster 3 was characterized by high levels of L-tryptophan, Homoveratric acid, Ricinoleic acid, Phloretin, Gluconolactone, trans-cinnamic acid, and 5-methoxysalicylic acid than in the other groups. Cluster 2 exhibited intermediate metabolite abundances, with balanced abundance of compounds enriched in Cluster 1 and Cluster 3.

### 3.4 Consistent directional shifts in metabolite relative abundances post-fermentation

As shown in Figure 4A, multiple functional metabolites displayed consistent upward trends with increasing wheat bran dosage, temperature, and fermentation time. The relative abundances of Tryptophan, Phenyllactic acid, Indolelactic acid, 4-Hydroxyphenyllactic acid, L-isoleucyl-L-proline, N-lactoyl-Phenylalanine, L-phenylalanyl-L-proline, trans-cinnamic acid, 5-methoxysalicylic acid, Homoveratric acid, Porphobilinogen, and 2,3-Dihydroxybenzoic acid were lowest in Group 0, gradually increased in Groups 1–3, and were highest in Groups 4–8 (FDR-adjusted *P* < 0.05).

**FIGURE 4:**
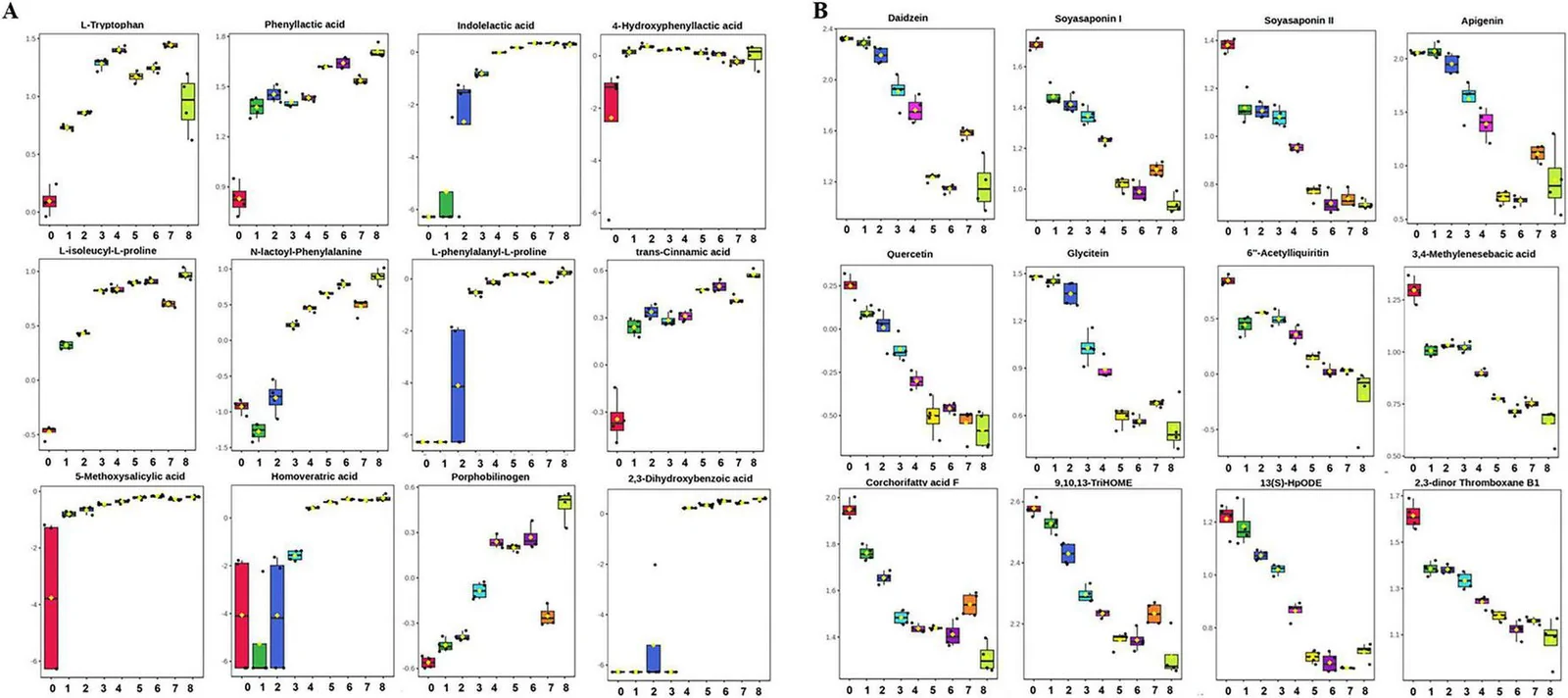
Metabolites displaying consistent directional abundance shifts along the fermentation parameter gradient. **(A)** Metabolites with significantly elevated concentrations with rising wheat bran supplementation, temperature, and fermentation duration (FDR-adjusted *P* < 0.05). **(B)** Metabolites with significantly reduced concentrations under intensified fermentation parameters (FDR-adjusted *P* < 0.05).

As shown in Figure 4B, the levels of Daidzin, Soyasaponin I, Soyasaponin II, Apigenin, Quercetin, Glycitein, 6”-Acetylliquiritin, 3,4-Methylenesebacic acid, Corchorifatty acid F, 9,10,13-TriHOME, 13(S)-HpODE, and 2,3-dinor Thromboxane B1 were highest in Group 0, followed by Groups 1–3, and lowest in Groups 4–8 (FDR-adjusted *P* < 0.05).

### 3.5 Relative explanatory power of substrate, temperature, and duration on microbial community structure and metabolite distributions

RDA analysis at the genus level (Figure 5A) indicated that significant differences were observed in the microbial community among different groups (*P* = 0.002). Gradual increases in wheat bran dosage, fermentation temperature, and incubation duration drove a directional community shift. The microbial structure of soybean residue was shifted from a community dominated by unclassified Enterobacteriaceae, unclassified Enterobacterales, unclassified Nostocaceae, *Enterobacter*, *Leuconostoc*, *Lactococcus*, *Pseudomonas*, and *Pantoea*, to a structure centered on genera *Limosilactobacillus*, *Lactiplantibacillus*, *Lacticaseibacillus*, unclassified Nitriliruptoraceae, *Achromobacter*, and *Bacillus*.

**FIGURE 5:**
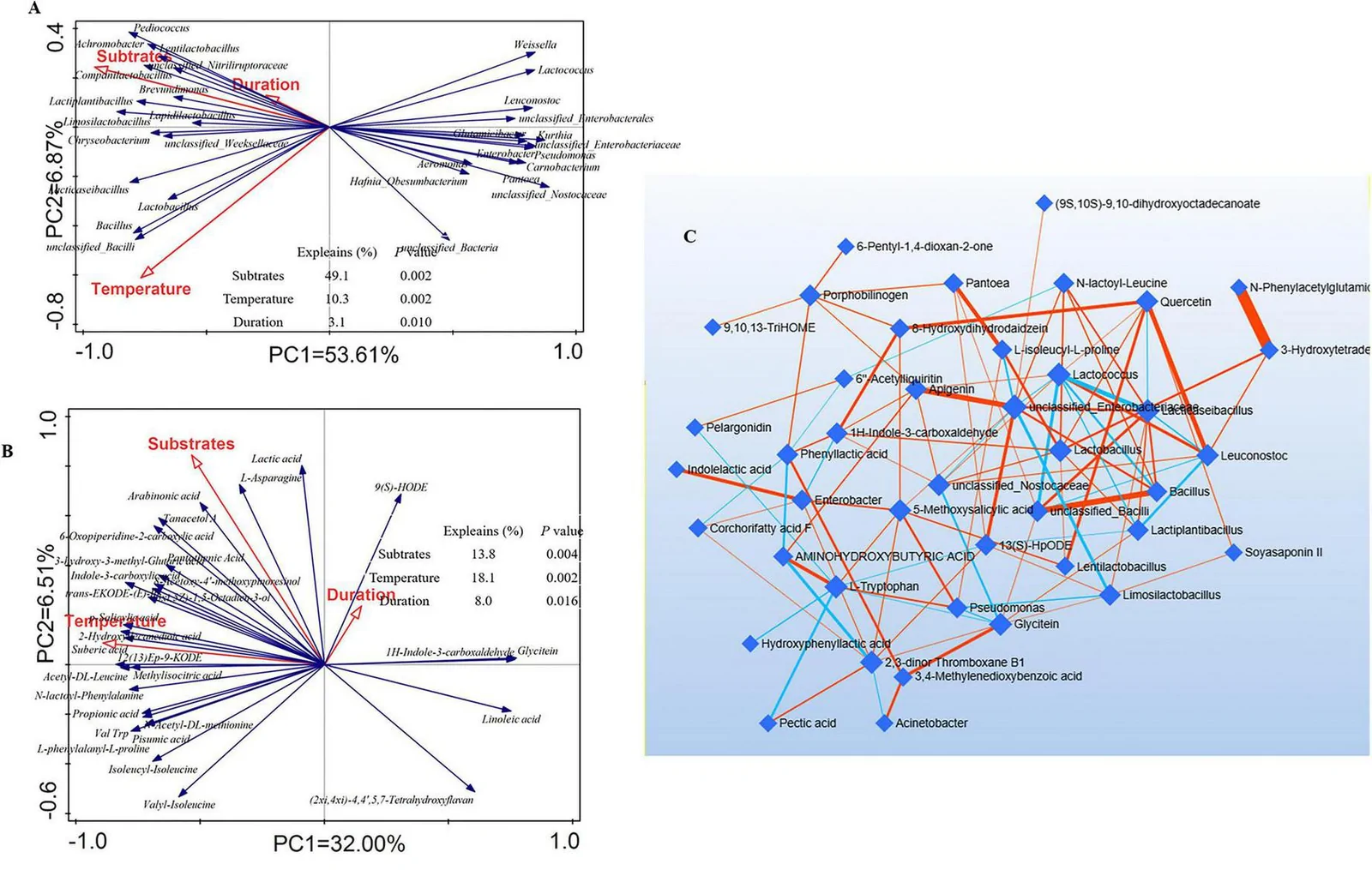
Independent effects of co-fermented material, temperature, duration on microbiota and metabolites, and their correlation networks in fermented soybean residue. **(A)** RDA analysis quantifying the independent explanatory power of three fermentation factors on genus-level microbial community variation. **(B)** RDA analysis illustrating the impacts of substrate, temperature, and duration on global metabolite profiles. **(C)** DSPC partial correlation network integrating 15 significantly differential bacterial genera and 27 core altered metabolites. Line thickness indicates the magnitude of partial correlation coefficients; red lines denote positive correlations, and blue lines denote negative correlations.

The relative individual contributions of each factor were assessed by variance partitioning of RDA models, which quantified the marginal (unique) variance explained by each variable while controlling for the others. The explanatory weight for substrate addition was found to be 49.1% of axes contributions, indicating a highly significant effect on microbial community distribution (*P* = 0.002); the weight for fermentation temperature was found to be 10.3% of axes contributions, also showing a significant effect on microbial community distribution (*P* = 0.002); fermentation time was found to have the lowest weight (3.1% of axes contributions) but still exerted a significant influence on microbial community distribution (*P* = 0.010). These results confirmed that substrate supplementation was the primary driver of microbial community succession, followed by fermentation temperature, with incubation duration exerting the weakest independent effect.

The RDA plot (Figure 5B) of metabolites revealed significant metabolite differentiation across all groups (*P* = 0.002). Elevated fermentation temperature corresponded to gradual reductions in Glycitein and 1H-indole-3-carboxaldehyde, alongside increasing abundances of Suberic acid, p-salicylic acid, Acetyl-DL-leucine, 2-hydroxydecanedioic acid, Methylisocitric acid, Propionic acid, and 12(13)-Ep-9-KODE. Rising wheat bran addition decreased levels of (2xi,4xi)-4,4’,5,7-tetrahydroxyflavan while enriching Arabinonic acid, Tanacetol A, L-asparagine, Lactic acid, 6-oxopiperidine-2-carboxylic acid, Pantothenic acid, and Indole-3-carboxylic acid. The relative abundance of 9(S)-HODE increased consistently with rising incubation duration.

Permutation-based variance partitioning identified fermentation temperature as the dominant factor structuring metabolite variation, explaining 18.1% of total axis contribution (*P* = 0.002). Substrate supplementation accounted for 13.8% of metabolite variance (*P* = 0.004), and fermentation duration explained 8.0% *(P* = 0.016). In contrast to microbial community assembly, metabolite distribution was primarily governed by incubation temperature rather than substrate addition.

### 3.6 Correlation between microbial communities and metabolites

A DSPC partial correlation network integrating 15 differential bacterial genera and 27 significantly altered metabolites was constructed to visualize microbial–metabolite interactions (Figure 5C). Pre-fermentation dominant taxa (unclassified Enterobacteriaceae, *Lactococcus*) and post-fermentation enriched probiotic genera (*Limosilactobacillus*, *Lactiplantibacillus*, *Lacticaseibacillus*, and *Lactobacillus*) exhibited strong partial correlations with global metabolite shifts. Key metabolites driving profile changes included L-tryptophan, glycitein, 2,3-dinor-thromboxane B1, 5-methoxysalicylic acid, quercetin, and aromatic lactic acid metabolites such as indole-3-carboxaldehyde, indolelactic acid, phenyllactic acid (PLA), and 4-hydroxyphenyllactic acid (4HPLA).

Unclassified Enterobacteriaceae was strongly correlated with the metabolism of (9S,10S)-9,10-dihydroxyoctadecanoate, 13(S)-HpODE, 5-methoxysalicylic acid, apigenin, L-isoleucyl-L-proline, and N-lactoyl-leucine. *Lacticaseibacillus* displayed tight correlations with 13(S)-HpODE and glycitein biosynthesis. *Lactococcus* co-varied significantly with apigenin and quercetin abundance, alongside the relative abundances of *Lacticaseibacillus*, unclassified Enterobacteriaceae, unclassified Bacilli, and unclassified Nostocaceae. *Lactobacillus*, unclassified Nostocaceae, *Pseudomonas*, and *Enterobacter* were closely associated with L-tryptophan and its downstream metabolites (indole-3-carboxaldehyde, indolelactic acid). *Limosilactobacillus* was strongly linked to the biotransformation of soybean-specific phytochemicals including glycitein, soyasaponin II, and 8-hydroxydihydrodaidzein.

## 4 Discussion

This study demonstrated that wheat bran supplementation during soybean residue fermentation triggers a fundamental reorganization of the bacterial community, shifting dominance from the phylum Proteobacteria to Firmicutes. This structural transition featured targeted enrichment of *Bacillus* and multiple lactic acid bacterial taxa, concurrent with the suppression of opportunistic pathogenic groups such as Enterobacteriaceae and *Pseudomonas*. These substrate-mediated microbial changes further remodeled the global metabolome, significantly elevating the levels of bioactive functional metabolites including L-tryptophan and indolelactic acid.

### 4.1 Microbial succession patterns in the fermentation of soybean residue

Successful solid-state feed fermentation relies on competitive interactions between indigenous microbes and artificially inoculated starter strains. Since raw substrates were not sterilized prior to inoculation, the fermentation ecosystem inherently contained native microbiota dominated by Proteobacteria, which constitute the predominant indigenous bacterial phylum in raw soybean materials (Tran et al., 2024) and are also common in soybean residue (Lian et al., 2019). The high protein and moisture content of raw soybean residue enables resident proteolytic *Proteobacteria* to decompose macromolecular proteins and release ammonia byproducts (Özogul and Özogul, 2007; Soliman and Eldyasti, 2018), which degrade nutritional value and accelerate feed spoilage (Driehuis and Oude Elferink, 2000). Following fermentation, the inoculated bacterial strains, including members from genera *Limosilactobacillus*, *Lactiplantibacillus*, *Lacticaseibacillus*, and *Bacillus*, became the predominant microbiota in the fermented soybean residue. The dominance of inoculated strains indicates successful colonization and competitive enrichment, rather than absolute exclusion of all indigenous taxa. Numerous studies have demonstrated that the predominance of inoculated strains within the fermentation community serves as both an indicator of successful inoculation and a key criterion for evaluating fermentation efficacy (Carvalho et al., 2021; Guo et al., 2022; Li et al., 2019). Concurrently, the relative abundances of genera *Lactobacillus*, unclassified Nitriliruptoraceae, and *Achromobacter* also increased, suggesting that the starter culture further drove the evolution of the overall dominant microbial community, thus further determines the fermentation pattern (Ke et al., 2025; Tahir et al., 2023). Notably, *Lactobacillus*-related bacterial taxa are widely recognized as probiotics, whereas Enterobacteriaceae-related taxa are considered potentially harmful microorganisms (Baldelli et al., 2021; Paterson, 2006). Once probiotic populations establish numerical dominance within the fermentation matrix, they exert comprehensive regulatory effects on fermentation pH, nutritional composition, and downstream metabolite profiles (Marco et al., 2017; Sharma et al., 2023).

### 4.2 Substrate supplementation as the primary driver of microbial community shifts, followed by temperature and durations

The co-fermented material exerted a larger independent effect on microbial community assembly than fermentation temperature or duration. As the fundamental carbon and nitrogen source for microbial metabolism, graded wheat bran addition (5–15%) represented the primary determinant of overall community structure and metabolite diversity in this study. Dry wheat bran contains low-abundance indigenous microbiota, including genera *Pantoea*, *Sphingomonas*, *Pseudomonas*, *Massilia*, and *Enterobacter* (Solanki et al., 2019; Solanki et al., 2021). However, genera *Pantoea*, *Sphingomonas*, *Massilia*, and *Erwinia* were not detected as dominant taxa, and both genera *Pseudomonas* and *Enterobacteriaceae* were significantly suppressed rather than enriched after fermentation. In contrast, a consistent and dose-dependent shift was observed toward genera from the fermentation inoculum, such as *Bacillus* and lactic acid bacteria (*Limosilactobacillus*, *Lactiplantibacillus*, *Lacticaseibacillus*) in wheat bran-supplemented groups (Anson et al., 2012). Furthermore, fermentation without wheat bran (Groups 1–3) still induced a clear shift from *Proteobacteria* to *Firmicutes*, confirming that inoculated strains can restructure the community independently of wheat bran microbiota. These observations indicate that the nutritional composition of wheat bran, rather than its epiphytic microbiome, was the primary driver of the observed microbial community shifts.

Although soybean residue contains abundant nutrients, such as dietary fiber and protein, its low soluble carbohydrate content limits the efficiency of microbial growth and metabolism (O’Toole, 1999). Wheat bran is rich in readily fermentable soluble saccharides and is routinely used as a supplementary carbon source in solid-state feed fermentation (El-Shishtawy et al., 2014), effectively compensating for the carbohydrate limitation of pure soybean residue. Bacterial strains exhibit inherent substrate preference and prioritize simple low-molecular-weight carbon sources over complex polysaccharides (Calderon et al., 2001). When readily degradable saccharides are available, microbes preferentially consume them and repress the expression of complex carbon catabolic pathways (Görke and Stülke, 2008). Simple carbohydrates promote the growth of lactic acid bacteria and facilitate the accumulation of lactic acid (Endo et al., 2011), which further reshaps community competitive dynamics and fermentation metabolic patterns (Temudo et al., 2008).

Fermentation temperature also significantly altered microbial community distributions, particularly enriching *Lactobacillus* populations (Hernández et al., 2019; Zotta et al., 2013). Temperature significantly affects the structure of the microbial community and the fermentation products in controlled fermentation systems (Wang et al., 2020), but does not alter the predominant microbiota in the community (Lee et al., 2014; Li et al., 2019). Although microbiota exhibit optimal growth and metabolic activity at their optimum temperature, many of them can also grow at higher or lower temperatures (Du et al., 2022), indicating that microorganisms possess a certain degree of temperature tolerance. However, the activity of microbial enzymes is highly sensitive to temperature (Di Luccio et al., 2004), which in turn modulates the overall metabolic activity of a given fermentation inoculum (Tu et al., 2025). This explains why temperature emerged as the primary factor regulating metabolite distribution in the present study.

Fermentation duration is widely recognized as a critical factor influencing microbial community structure and metabolite profiles (Hamdouche et al., 2019; Jeong et al., 2013). However, in the present study, fermentation durations ranging from 24 to 72 h did not yield consistent changes in either microbial communities or metabolic products. This suggests that the magnitude of temporal effects is strongly dependent on substrate availability and incubation temperature (Kim et al., 2006; Lv et al., 2019). Although fermentation duration can significantly affect the final products of microbial enzymatic activities (Di Stasio et al., 2025; Gänzle, 2014), our observations indicate that under suitable temperature and sufficient substrate availability, the microbial consortium can achieve optimal metabolic performance within a relatively short period. In contrast, insufficient substrate or inappropriate temperature requires prolonged fermentation to reach equivalent metabolite yields.

### 4.3 Flavonoid degradation, elevated phenolic acids, and aromatic amino acid biotransformation during fermentation

Microbial succession induced opposing abundance trends for flavonoids and phenolic acid derivatives in fermented soybean residue. Levels of flavonoid metabolites were found to decrease after the fermentation process. Flavonoid metabolites retain structural and functional similarities to their parent compounds and exhibit comparable bioactivity during animal digestion and absorption (Gonzales et al., 2015). Microbes mediate the catabolism of flavonoids in flavonoid-rich co-fermented materials, driving measurable reductions in total flavonoid concentrations (Simons et al., 2005). In the fermentation of material rich in flavonoids, the microbial community has been observed to play a significant role in the catabolism of flavonoids, resulting in a decline in these flavonoid metabolites (Lee et al., 2019; Li et al., 2024). The initial degradation step involves microbial β-glucosidase-mediated hydrolysis of flavonoid glycosides into aglycone forms, which are further degraded into secondary metabolites such as dihydrosoyaglycone (Tsuchihashi et al., 2008).

The levels of phenolic acid metabolites, which were originally low in soybean residue, increased significantly after fermentation. This phenomenon has been attributed to the action of microbial hydrolytic enzymes, which cleave bound phenolic complexes to release free phenolic monomers (Liu et al., 2026). Free phenolic compounds are subsequently modified by microbial esterases, decarboxylases, and reductases to generate novel phenolic acid derivatives with enhanced biological activity (Kumar et al., 2025), which deliver substantial benefits to livestock intestinal health.

Three aromatic amino acids (tryptophan, phenylalanine, tyrosine) produce characteristic aromatic lactic acid metabolites: indolelactic acid (ILA), phenyllactic acid (PLA), and 4-hydroxyphenyllactic acid (4HPLA), which displayed coordinated accumulation across fermentation treatments. Soybean matrix proteins are gradually degraded by microbial proteases to release free and polymeric aromatic amino acids (Frias et al., 2008; Han et al., 2004). Aromatic amino acids are initially converted via a process known as transamination, which results in the formation of the corresponding aromatic pyruvic acids (Laursen et al., 2021). This reaction has been identified as a pivotal node common to all three pathways, with the catalyzation of this process being facilitated by a single enzyme (Dodd et al., 2017). ILA, PLA, and 4HPLA exert synergistic regulatory effects on animal intestinal barrier integrity and immune homeostasis via aryl hydrocarbon receptor (AhR) (Hu et al., 2023; Scott et al., 2020) and hydroxycarboxylic acid receptor 3 (HCA3) (Peters et al., 2019). ILA strengthens intestinal epithelial barrier function and suppresses mucosal inflammation by stimulating IL-22 secretion (Laursen et al., 2021; Wang et al., 2025). PLA elevates peripheral lymphocyte counts in livestock, and combined PLA + 4HPLA supplementation alleviates hepatic steatosis induced by high-fat diets while modulating macrophage polarization (Lim et al., 2025). The consistent enrichment of aromatic amino acids and their bioactive derivatives in wheat bran supplemented fermented soybean residue provides a mechanistic basis for improved animal immunity and intestinal health when this material is incorporated into feed formulations.

We acknowledge that wheat bran has a distinct nutritional profile and that a raw mixture control would further clarify the origin of the observed metabolite changes. However, the amounts of aromatic lactic acids are relatively low in wheat bran protein (CFDI, 2024), and high phytate content in wheat bran forms insoluble complexes with free amino acids, further reducing bioavailable protein in raw sterilized bran substrate (Selle et al., 2012). Wheat bran is known to contain bound phenolic acids such as ferulic acid, p-coumaric acid, and vanillic acid (Anson et al., 2012; Tiwari et al., 2025), raw wheat bran only accumulates simple phenolic acids (ferulic acid, p-coumaric acid), but not the aromatic lactic acids. The linear accumulation of target aromatic metabolites along wheat bran supplementation gradients (0–15%) in fermented groups solely arises from boosted proteolytic activity of inoculated lactic acid bacteria stimulated by bran soluble carbohydrates, rather than inherent aromatic amino acid pools supplied by wheat bran itself. However, we acknowledge that future studies incorporating metabolomic analysis of unfermented substrate mixtures and sterile fermentation controls would provide more definitive evidence to fully resolve the relative contributions of substrate-derived compounds versus microbial metabolic activities.

We acknowledge that the absence of proximate nutritional analysis (crude protein, fiber fractions, total organic acids) and antinutritional factor quantification limited the direct assessment of fermentation performance. While the metabolomic and microbiome data provide mechanistic insights into biotransformation pathways, absolute quantification of nutritional and anti-nutritional parameters is required to fully evaluate feed quality. These analyses will be integrated in subsequent studies.

## 5 Conclusion

The present study confirms that wheat bran substrate supplementation drives profound structural shifts in the bacterial community of fermented soybean residue, selectively enriching beneficial *Bacillus* and lactic acid bacterial genera while suppressing opportunistic pathogenic Enterobacteriaceae and *Pseudomonas*. These microbial alterations reshape the global metabolome, significantly elevating levels of functional bioactive metabolites including L-tryptophan and indolelactic acid, while reducing total flavonoid levels (Figure 6). These findings lay the groundwork for optimizing fermentation conditions, providing valuable insights into microbial and metabolite process for feed production applications, and supporting the sustainable development of livestock production by improving the utilization of soybean processing by-products.

**FIGURE 6:**
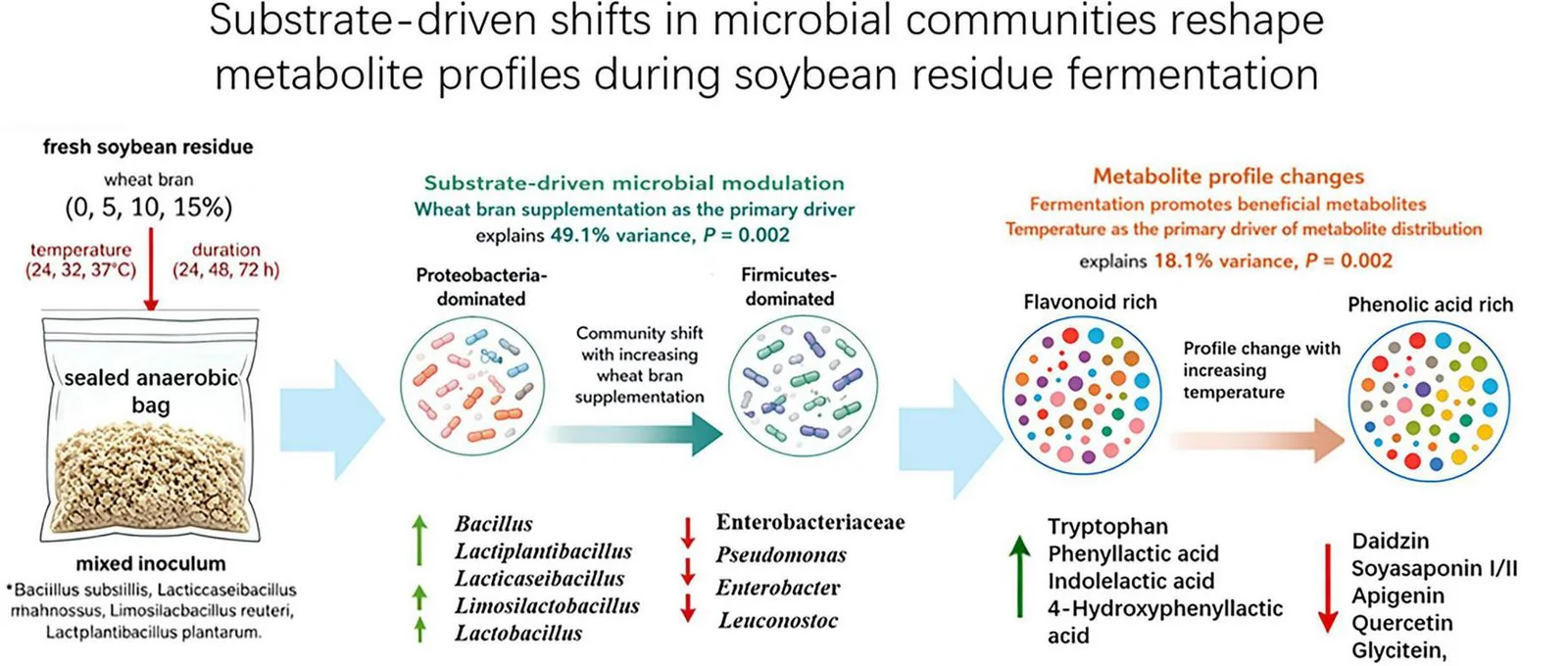
The schematic diagram of experimental design and main results in this study.

## Data Availability

The raw sequencing data have been deposited in the NCBI Sequence Read Archive (SRA) under BioProject accession number PRJNA1478431.

## Footnotes

1
http://www.hmdb.ca/

2
http://www.massbank.jp/

3
http://metlin.scripps.edu/

4
https://www.metaboanalyst.ca/

## Supplementary material

Supplementary Figure S1 Comparison fo pH values between unfermented soybean residue (Group 0) and fermented treatment groups at the end point of fermentation (Group 1-8).
