# Supplementary material for: Substrate-driven shifts in microbial communities reshape metabolite profiles during soybean residue fermentation
Source: Front Microbiol. 2026 Sep 28;17:1919307. doi: 10.3389/fmicb.2026.1919307 (PMC13654976; doi:10.3389/fmicb.2026.1919307)
Supplement: Supplementary file 1 [file Supplementary_file_1.docx]

**Supplementary Figure Legend**

**Figure S1** Comparison of pH values between unfermented soybean residue (Group 0) and fermented treatment groups at the end piont of fermentation (Groups 1–8). Different lowercase letters indicate significant differences among groups (FDR-adjusted P < 0.05).
