# Supplementary material for: Substrate-driven shifts in microbial communities reshape metabolite profiles during soybean residue fermentation
Source: Front Microbiol. 2026 Sep 28;17:1919307. doi: 10.3389/fmicb.2026.1919307 (PMC13654976; doi:10.3389/fmicb.2026.1919307)
Supplement: Supplementary file 2 [file Table_1.docx]

**Table S1 Comprehensive list of identified metabolites with m/z, retention time, MS/MS fragmentation score, and isotope similarity**

| m/z | Retention time (min) | Chromatographic peak width (min) | Identifications | Isotope Distribution | Accepted Compound ID | Accepted Description | Formula | Score | Fragmentation Score | Mass Error (ppm) | Isotope Similarity |
| --- | --- | --- | --- | --- | --- | --- | --- | --- | --- | --- | --- |
| 373.1301 | 7.608283 | 0.125617 | 7 | 100 - 12.7 | HMDB30598 | (-)-Wikstromol | C20H22O7 | 42.5 | 25.8 | 2.156523 | 89.16547 |
| 283.2633 | 11.8625 | 0.385217 | 6 | 100 - 18.2 - 0.55 | 4293 | (+)-Isostearic acid | C18H36O2 | 41.1 | 12.3 | -3.28948 | 96.96675 |
| 203.129 | 6.64515 | 0.192617 | 5 | 100 - 6.99 | HMDB39053 | (1alpha,2alpha,4betaH,6alpha,8R)-p-Menthane-2,6,8,9-tetrol | C10H20O4 | 42.8 | 20 | 0.68548 | 94.93426 |
| 291.1967 | 10.84918 | 0.1926 | 6 | 100 - 13.3 | HMDB30996 | (2'E,4'Z,7'Z,8E)-Colnelenic acid | C18H28O3 | 46.4 | 40.2 | 0.511365 | 92.39561 |
| 271.0615 | 7.35705 | 0.217733 | 18 | 100 - 7.28 | HMDB37254 | (2R,3R)-3,4',7-Trihydroxyflavanone | C15H12O5 | 40.9 | 15.6 | 1.138349 | 90.08505 |
| 273.0775 | 7.532917 | 0.142367 | 25 | 100 - 7.04 | HMDB37232 | (2xi,4xi)-4,4',5,7-Tetrahydroxyflavan | C15H14O5 | 43.4 | 29.7 | 2.339235 | 89.85501 |
| 125.0972 | 7.264933 | 0.117233 | 24 | 100 - 6.8 | HMDB30966 | (3xi,5Z)-1,5-Octadien-3-ol | C8H14O | 45.1 | 28 | 0.234701 | 97.61789 |
| 315.2543 | 10.44722 | 0.15075 | 4 | 100 - 16.9 | HMDB59633 | (9S,10S)-9,10-dihydroxyoctadecanoate | C18H36O4 | 45.4 | 32.9 | 0.603046 | 94.97956 |
| 309.2073 | 9.71865 | 0.527583 | 8 | 100 - 20.7 | HMDB33505 | (9Z,11R,12S,13S,15Z)-12,13-Epoxy-11-hydroxy-9,15-octadecadienoic acid | C18H30O4 | 52 | 63.9 | 0.521081 | 96.95596 |
| 295.2271 | 11.38515 | 0.351733 | 18 | 100 - 19.3 | 43442 | (β)12(13)-EpOME | C18H32O3 | 44.5 | 28.1 | -2.46263 | 97.27228 |
| 313.2386 | 9.559533 | 0.3601 | 7 | 100 - 15.8 | 45199 | (β)12,13-DiHOME | C18H34O4 | 43.7 | 25.2 | 0.552578 | 94.11606 |
| 173.1185 | 7.35705 | 0.159117 | 2 | 100 - 9.07 | HMDB31513 | (β)-3-Hydroxynonanoic acid | C9H18O3 | 42.7 | 16.8 | 1.060615 | 98.15179 |
| 311.2229 | 11.10042 | 0.100483 | 13 | 100 - 11.9 | 64785 | (β)9-HpODE | C18H32O4 | 48.7 | 53.3 | 0.433062 | 90.96019 |
| 395.153 | 10.95805 | 0.134 | 10 | 100 - 18.5 - 1 | HMDB39953 | 1-(3-Methylbutanoyl)-6-apiosylglucose | C16H28O11 | 47 | 46.2 | -7.30192 | 96.95359 |
| 149.0246 | 7.8009 | 0.117233 | 5 | 100 - 0.683 | HMDB40528 | 1,4-Benzodioxin-2(3H)-one | C8H6O3 | 45.3 | 36.5 | 1.295379 | 91.70003 |
| 269.2118 | 11.32653 | 0.092117 | 4 | 100 - 12.2 | HMDB30978 | 11-Oxohexadecanoic acid | C16H30O3 | 43.8 | 27.3 | -1.53691 | 93.45391 |
| 309.2072 | 11.02505 | 0.192617 | 8 | 100 - 13.2 | HMDB13623 | 12(13)Ep-9-KODE | C18H30O4 | 50.2 | 59.6 | 0.323706 | 92.04695 |
| 311.223 | 9.752133 | 0.44385 | 13 | 100 - 19.2 | 36036 | 13(S)-HpODE | C18H32O4 | 51.6 | 61.7 | 0.573783 | 96.96209 |
| 357.2648 | 9.760517 | 0.08375 | 2 | 100 - 17.4 | HMDB05076 | 13,14-Dihydro PGF-1a | C20H38O5 | 44.8 | 31.5 | 0.473789 | 93.17876 |
| 299.2581 | 11.64477 | 0.242867 | 7 | 100 - 18.9 - 1.01 | HMDB61662 | 13-hydroxyoctadecanoic acid | C18H36O3 | 44 | 26.4 | -3.43109 | 97.70606 |
| 144.0456 | 7.348683 | 0.167483 | 2 | 100 - 9.46 | HMDB29737 | 1H-Indole-3-carboxaldehyde | C9H7NO | 42.6 | 15.3 | 0.864066 | 98.70496 |
| 161.0822 | 3.20325 | 0.44385 | 2 | 100 - 5.96 - 0.0108 | HMDB59918 | 2-(1-Ethoxyethoxy)propanoic acid | C7H14O4 | 43.3 | 21.3 | 1.677108 | 97.23972 |
| 153.0194 | 7.013617 | 0.4858 | 10 | 100 - 4.2 - 0.0277 | 3276 | 2,3-Dihydroxybenzoic acid | C7H6O4 | 48.2 | 45.9 | 0.301601 | 95.73392 |
| 325.2027 | 7.909767 | 0.3601 | 4 | 100 - 17.9 | 45460 | 2,3-dinor Prostaglandin E1 | C18H30O5 | 47.5 | 44.1 | 2.014729 | 95.65098 |
| 343.213 | 7.3738 | 0.561167 | 2 | 100 - 20.5 | 44923 | 2,3-dinor Thromboxane B1 | C18H32O6 | 40.5 | 7.29 | 1.26293 | 96.78668 |
| 183.0049 | 8.5211 | 0.184233 | 1 | 100 | 43994 | 2,4-DINITROPHENOL | C6H4N2O5 | 41.2 | 15.1 | 0.744504 | 91.97466 |
| 131.0714 | 6.243167 | 0.519217 | 21 | 100 - 5.87 - 0.105 | 6411 | 2-Ethyl-2-Hydroxybutyric acid | C6H12O3 | 52 | 61.8 | 0.383186 | 98.53687 |
| 117.0558 | 3.404233 | 0.452217 | 17 | 100 - 1.53 - 0.00968 | 45847 | 2-hydroxy-2-methyl-butyric acid | C5H10O3 | 43.1 | 21.3 | 0.937082 | 95.4505 |
| 217.1082 | 6.770767 | 0.133983 | 2 | 100 - 8.97 | HMDB00424 | 2-Hydroxydecanedioic acid | C10H18O5 | 44.2 | 25.1 | 0.428804 | 96.48203 |
| 271.228 | 11.1088 | 0.142367 | 6 | 100 - 2.59 | 34495 | 2-hydroxyhexadecanoic acid | C16H32O3 | 42.6 | 28.2 | 0.544184 | 85.43655 |
| 331.1189 | 6.728883 | 0.167483 | 9 | 100 - 18.2 - 0.223 | HMDB30664 | 3,3'-Dihydroxy-4',5,7-trimethoxyflavan | C18H20O6 | 42.7 | 18.3 | 0.660499 | 95.94451 |
| 165.0194 | 8.253117 | 0.150733 | 4 | 100 - 4.4 - 0.0353 | HMDB32613 | 3,4-Methylenedioxybenzoic acid | C8H6O4 | 42.5 | 18 | 0.309633 | 94.90621 |
| 225.1133 | 6.368783 | 0.234483 | 2 | 100 - 12.5 | HMDB59729 | 3,4-Methylenesebacic acid | C12H18O4 | 44.4 | 25 | 0.416743 | 97.70271 |
| 179.0557 | 1.168267 | 0.41035 | 35 | 100 | HMDB00346 | 3-Deoxyarabinohexonic acid | C6H12O6 | 44.2 | 31.4 | -2.26288 | 92.34558 |
| 161.0451 | 1.252017 | 0.27635 | 11 | 100 - 2.57 | 3793 | 3-hydroxy-3-methyl-Glutaric acid | C6H10O5 | 49.7 | 57.1 | -2.86324 | 94.95475 |
| 245.1396 | 7.197933 | 0.134 | 2 | 100 - 10.5 | HMDB00413 | 3-Hydroxydodecanedioic acid | C12H22O5 | 47.2 | 41.1 | 0.592843 | 95.72838 |
| 145.0872 | 7.465917 | 0.167483 | 7 | 100 - 6.98 | HMDB02207 | 3-Hydroxyisoheptanoic acid | C7H14O3 | 44.5 | 26 | 1.536318 | 98.39505 |
| 273.1712 | 7.432417 | 0.167483 | 1 | 100 - 7.09 | HMDB00394 | 3-Hydroxytetradecanedioic acid | C14H26O5 | 43.2 | 27.2 | 1.82081 | 90.74847 |
| 174.0563 | 7.549667 | 0.133983 | 5 | 100 - 1.46 | 70 | 3-Indoleacetic Acid | C10H9NO2 | 45.1 | 37.4 | 1.63007 | 90.29975 |
| 206.0462 | 3.13625 | 0.209367 | 2 | 100 - 0.974 | HMDB00978 | 4-(2-Aminophenyl)-2,4-dioxobutanoic acid | C10H9NO4 | 41.5 | 20 | 1.485898 | 89.38407 |
| 180.0667 | 6.343667 | 0.159117 | 11 | 100 - 2.7 | HMDB01119 | 4-Hydroxy-4-(3-pyridyl)-butanoic acid | C9H11NO3 | 45.7 | 36.6 | 0.214655 | 92.15619 |
| 292.1031 | 1.653983 | 0.083733 | 4 | 100 - 0.497 | HMDB29354 | 4-Hydroxyproline galactoside | C11H19NO8 | 42.3 | 27.3 | -2.2977 | 87.07161 |
| 119.0502 | 6.728883 | 0.217733 | 9 | 100 - 8.23 | HMDB04072 | 4-Hydroxystyrene | C8H8O | 43.7 | 19.6 | -0.03373 | 98.99822 |
| 138.0199 | 7.85115 | 0.200983 | 6 | 100 - 5.59 - 0.0923 | 4100 | 4-Nitrophenol | C6H5NO3 | 53.9 | 73 | 1.36848 | 97.98589 |
| 116.0713 | 1.151517 | 0.1926 | 7 | 100 - 0.0997 | HMDB03355 | 5-Aminopentanoic acid | C5H11NO2 | 46.4 | 42.4 | -3.61881 | 93.95899 |
| 305.1758 | 9.61815 | 0.050233 | 2 | 100 - 17.6 | HMDB12799 | 5'-Carboxy-gamma-chromanol | C18H26O4 | 43.1 | 19.8 | -0.00696 | 95.65684 |
| 167.035 | 5.9082 | 0.20935 | 19 | 100 - 4.75 | 4164 | 5-Methoxysalicylic acid | C8H8O4 | 51.2 | 61.4 | 0.303177 | 95.16744 |
| 459.13 | 6.410667 | 0.167483 | 4 | 100 - 26.1 - 1.75 | HMDB41385 | 6''-Acetylliquiritin | C23H24O10 | 47.5 | 41.8 | 0.730593 | 96.35961 |
| 465.2095 | 8.86445 | 0.08375 | 3 | 100 - 22 | HMDB60797 | 6alpha,9alpha-Difluoroprednisolone-17-butyrate | C25H32F2O6 | 40.5 | 11.2 | 0.088291 | 91.59631 |
| 241.181 | 9.87775 | 0.134 | 3 | 100 - 9.75 | HMDB30982 | 6-Ketomyristic acid | C14H26O3 | 43 | 22.3 | 0.498877 | 93.44428 |
| 142.0504 | 1.226883 | 0.108867 | 5 | 100 - 1.14 | HMDB61705 | 6-Oxopiperidine-2-carboxylic acid | C6H9NO3 | 45.5 | 38 | -3.66478 | 93.73281 |
| 171.1029 | 8.027 | 0.293117 | 9 | 100 - 7.86 - 0.106 | HMDB59937 | 6-Pentyl-1,4-dioxan-2-one | C9H16O3 | 45.7 | 32.6 | 1.095367 | 97.17552 |
| 429.1588 | 9.006817 | 0.318233 | 3 | 100 - 16.8 - 2.99 | HMDB33277 | 8-Acetoxy-4'-methoxypinoresinol | C23H26O8 | 41.6 | 25.1 | 7.777048 | 91.52828 |
| 271.0619 | 8.253117 | 0.267967 | 18 | 100 - 15.8 | HMDB41704 | 8-Hydroxydihydrodaidzein | C15H12O5 | 49.8 | 54.6 | 2.502276 | 97.26042 |
| 293.212 | 11.15905 | 0.5276 | 25 | 100 - 19 | HMDB10220 | 9(10)-EpODE | C18H30O3 | 54.5 | 76.4 | -0.71087 | 97.03096 |
| 295.2279 | 10.95805 | 0.561083 | 18 | 100 - 20.9 | 45662 | 9(S)-HODE | C18H32O3 | 51.3 | 59.7 | 0.244598 | 96.99021 |
| 293.2124 | 10.62308 | 0.351733 | 25 | 100 - 19.1 | 36030 | 9(S)-HOTrE | C18H30O3 | 54.3 | 75.3 | 0.626517 | 97.0971 |
| 309.2073 | 10.6147 | 0.1926 | 8 | 100 - 18.4 | 36025 | 9(S)-HpOTrE | C18H30O4 | 52.3 | 66.1 | 0.597091 | 96.28989 |
| 329.2341 | 8.4206 | 0.418717 | 4 | 100 - 19.6 - 1.8 | HMDB04710 | 9,10,13-TriHOME | C18H34O5 | 49.5 | 51.6 | 2.129046 | 98.42882 |
| 331.2491 | 8.671833 | 0.334983 | 2 | 100 - 19.6 - 0.929 | HMDB30935 | 9,10,13-Trihydroxystearic acid | C18H36O5 | 45.7 | 31.1 | 0.278098 | 97.73448 |
| 311.2229 | 9.232933 | 0.2931 | 13 | 100 - 13.9 | HMDB10221 | 9,10-DiHODE | C18H32O4 | 46.4 | 39.8 | 0.463753 | 92.58456 |
| 293.2124 | 9.844267 | 0.058617 | 25 | 100 - 5.95 | HMDB10224 | 9-HOTE | C18H30O3 | 42.6 | 27.8 | 0.77913 | 86.38158 |
| 363.2154 | 10.91618 | 0.184233 | 8 | 100 - 15 | 263546 | Acetoxy-8-gingerol | C21H32O5 | 43.2 | 32.7 | -6.43384 | 90.36408 |
| 172.098 | 5.983567 | 0.217733 | 8 | 100 - 2.08 | 73549 | Acetyl-DL-Leucine | C8H15NO3 | 49.1 | 53.5 | 0.37062 | 92.54467 |
| 134.0468 | 1.277133 | 0.159117 | 3 | 100 - 4.09 | 85 | Adenine | C5H5N5 | 47.3 | 42.9 | -2.83547 | 96.80605 |
| 235.1093 | 2.5668 | 0.334967 | 6 | 100 - 0.797 | HMDB28694 | Alanyl-Phenylalanine | C12H16N2O3 | 41.8 | 24 | 1.968179 | 87.15253 |
| 199.0979 | 7.591533 | 0.125617 | 8 | 100 - 2.76 | HMDB30990 | alpha-Carboxy-delta-nonalactone | C10H16O4 | 43.4 | 27.6 | 1.399426 | 91.22635 |
| 218.0822 | 6.762383 | 0.159117 | 4 | 100 - 0.959 | HMDB38338 | alpha-Hydroxy-1-methyl-1H-indole-3-propanoic acid | C12H13NO3 | 41.5 | 20.2 | -0.0997 | 87.64132 |
| 537.0841 | 7.918133 | 0.08375 | 4 | 100 - 32.3 | HMDB30832 | Amentoflavone | C30H18O10 | 44.2 | 30.9 | 2.652938 | 93.295 |
| 130.0869 | 1.176633 | 0.11725 | 13 | 100 - 2.14 - 0.0951 | HMDB01901 | Aminocaproic acid | C6H13NO2 | 41 | 13.5 | -3.14334 | 94.92476 |
| 118.0507 | 1.084517 | 0.17585 | 7 | 100 - 0.108 - 0.034 | 44197 | AMINOHYDROXYBUTYRIC ACID | C4H9NO3 | 51.3 | 64.1 | -2.14336 | 94.81133 |
| 269.0458 | 8.378733 | 0.452217 | 19 | 100 - 17.4 | HMDB02124 | Apigenin | C15H10O5 | 48.3 | 45.8 | 1.106511 | 97.17846 |
| 563.1411 | 6.109183 | 0.276367 | 9 | 100 - 29.9 - 2.47 | HMDB30843 | Apiin | C26H28O14 | 53.9 | 75.6 | 0.8231 | 95.09241 |
| 165.04 | 1.151517 | 0.175867 | 6 | 100 - 5.07 - 0.117 | HMDB00539 | Arabinonic acid | C5H10O6 | 47 | 40 | -2.83738 | 98.19933 |
| 287.0566 | 7.759033 | 0.167483 | 7 | 100 - 11.4 | HMDB30847 | Aromadendrin | C15H12O6 | 47.7 | 47.5 | 1.815 | 93.30461 |
| 175.0243 | 1.218517 | 0.34335 | 5 | 100 - 5.57 - 0.593 | HMDB00044 | Ascorbic acid | C6H8O6 | 49.6 | 53.6 | -2.99373 | 98.08028 |
| 244.1304 | 1.737733 | 0.318233 | 8 | 100 - 5.77 | HMDB28734 | Asparaginyl-Isoleucine | C10H19N3O4 | 47.9 | 47.6 | 0.674239 | 92.84548 |
| 269.0882 | 1.7126 | 0.2931 | 3 | 100 - 8.1 | HMDB28755 | Aspartyl-Histidine | C10H14N4O5 | 41.6 | 17.6 | -3.32302 | 94.37238 |
| 187.0977 | 7.264933 | 0.184233 | 11 | 100 - 9.89 | HMDB00784 | Azelaic acid | C9H16O4 | 48.9 | 46.5 | 0.629927 | 98.69532 |
| 427.1434 | 8.1945 | 0.08375 | 2 | 100 - 25.7 | HMDB41221 | b-D-Xylopyranosyl-(1->4)-a-L-rhamnopyranosyl-(1->2)-L-arabinose | C16H28O13 | 47.3 | 52.5 | -5.44717 | 90.50707 |
| 121.0295 | 6.955 | 0.108867 | 6 | 100 - 0.523 | 1297 | Benzoic acid | C7H6O2 | 46 | 37.7 | 0.369647 | 92.74715 |
| 147.0454 | 1.930333 | 0.226117 | 9 | 100 - 0.253 - 0.0987 | HMDB00567 | Cinnamic acid | C9H8O2 | 42 | 21.3 | 1.464041 | 90.60997 |
| 289.0723 | 7.742283 | 0.1005 | 16 | 100 - 1.43 | HMDB32322 | cis-3 and trans-2-Hexenyl propionate | C15H14O6 | 46.6 | 50.3 | 1.829138 | 84.94906 |
| 307.1916 | 9.165933 | 0.159117 | 7 | 100 - 18.3 | HMDB33243 | Corchorifatty acid D | C18H28O4 | 50.2 | 55.4 | 0.298173 | 96.21019 |
| 327.218 | 8.17775 | 0.401967 | 3 | 100 - 19.3 | HMDB35919 | Corchorifatty acid F | C18H32O5 | 50.8 | 58.2 | 0.980377 | 96.77823 |
| 359.1833 | 2.84315 | 0.142367 | 9 | 100 - 8.38 | HMDB02802 | Cortisone | C21H28O5 | 41.7 | 33 | -8.50623 | 85.17482 |
| 211.1342 | 8.11075 | 0.150733 | 7 | 100 - 7.99 | HMDB29388 | Cucurbic acid | C12H20O3 | 42.3 | 18.6 | 1.001342 | 94.01019 |
| 199.9686 | 1.4195 | 0.175867 | 2 | 100 - 3.34 - 2.66 | 58010 | Cysteine-S-sulfate | C3H7NO5S2 | 48.1 | 53.5 | -3.34635 | 90.97971 |
| 253.0514 | 7.71715 | 0.318217 | 11 | 100 - 16.6 | 43572 | Daidzein | C15H10O4 | 48.6 | 48.3 | 2.991901 | 97.98083 |
| 159.0774 | 1.084517 | 0.150733 | 4 | 100 - 3.7 | 6937 | D-Ala-D-Ala | C6H12N2O3 | 48.3 | 46.8 | -0.96355 | 95.72351 |
| 149.0451 | 1.310633 | 0.150733 | 27 | 100 - 4.53 - 0.817 | HMDB29941 | D-Apiose | C5H10O5 | 45 | 29.9 | -2.70284 | 98.57268 |
| 677.4255 | 8.17775 | 0.117233 | 1 | 100 - 46.7 - 3.7 | HMDB60814 | Descladinose roxithromycin | C33H62N2O12 | 40.4 | 16.9 | 3.667149 | 89.39783 |
| 317.0672 | 7.516167 | 0.100483 | 6 | 100 - 3.65 | HMDB37501 | Dihydroisorhamnetin | C16H14O7 | 44.4 | 38.4 | 1.770922 | 85.6769 |
| 299.0563 | 7.315183 | 0.11725 | 21 | 100 - 10.8 | 44398 | Diosmetin | C16H12O6 | 50.6 | 62 | 0.729868 | 91.83155 |
| 229.1446 | 8.76395 | 0.142367 | 2 | 100 - 4.82 | HMDB00623 | Dodecanedioic acid | C12H22O4 | 45.1 | 34.6 | 0.235562 | 91.01031 |
| 276.0181 | 1.653983 | 0.201 | 3 | 100 | HMDB02028 | DOPA sulfate | C9H11NO7S | 43.7 | 35.1 | -0.90152 | 84.3084 |
| 147.0295 | 1.193383 | 0.20935 | 12 | 100 - 2.43 - 0.729 | HMDB11676 | D-Xylono-1,5-lactone | C5H8O5 | 46.2 | 38 | -2.93077 | 96.54488 |
| 255.0669 | 7.8009 | 0.24285 | 11 | 100 - 16.7 | HMDB36457 | Emodinanthranol | C15H12O4 | 51 | 59.8 | 2.414144 | 97.88945 |
| 135.0295 | 1.1599 | 0.167483 | 6 | 100 - 2.17 - 0.0191 | HMDB00613 | Erythronic acid | C4H8O5 | 44 | 27 | -3.04736 | 96.65857 |
| 193.0508 | 6.888 | 0.117233 | 16 | 100 - 5.92 | 4156 | ferulic acid | C10H10O4 | 49 | 52 | 0.711594 | 94.09488 |
| 433.1151 | 5.1545 | 0.108867 | 10 | 100 - 16 | HMDB33739 | Floribundoside | C21H22O10 | 45.2 | 38.7 | 2.516128 | 90.12345 |
| 367.248 | 11.25117 | 0.209367 | 7 | 100 - 16.9 | HMDB37961 | gamma-Eudesmol rhamnoside | C21H36O5 | 41.9 | 20.8 | -2.83436 | 91.81 |
| 259.1305 | 2.15645 | 0.27635 | 3 | 100 - 2.04 - 0.0171 | 23750 | Gamma-Glu-Leu | C11H20N2O5 | 47.3 | 50.2 | 2.01041 | 88.73041 |
| 225.077 | 6.670267 | 0.167483 | 8 | 100 - 10.1 | HMDB35830 | Genipin | C11H14O5 | 48.8 | 48.5 | 0.790487 | 96.46341 |
| 153.0194 | 5.698833 | 0.142367 | 10 | 100 - 4.52 | 618 | Gentisic acid | C7H6O4 | 45.2 | 30.8 | 0.549678 | 96.00173 |
| 195.0506 | 1.151517 | 0.150733 | 13 | 100 - 6.7 - 0.797 | 345 | Gluconic acid | C6H12O7 | 46.4 | 35.7 | -2.00748 | 98.95261 |
| 177.0401 | 1.653983 | 0.301483 | 11 | 100 - 3.12 - 0.351 | HMDB00150 | Gluconolactone | C6H10O6 | 49.7 | 55.5 | -2.07672 | 95.57198 |
| 131.0352 | 2.558417 | 0.34335 | 12 | 100 | 3254 | Glutaric acid | C5H8O4 | 46.2 | 39.1 | 1.559642 | 93.80449 |
| 245.1141 | 1.653983 | 0.150733 | 7 | 100 - 5.37 | HMDB59717 | Glu-Val | C10H18N2O5 | 42.9 | 23.3 | -0.97681 | 92.61571 |
| 353.1009 | 6.728883 | 0.1926 | 18 | 100 - 20.7 - 2.6 | HMDB34111 | Glyceofuran | C20H18O6 | 44 | 29.3 | -6.13234 | 97.83254 |
| 283.0617 | 7.809267 | 0.251233 | 24 | 100 - 17 | 47621 | Glycitein | C16H12O5 | 53.9 | 74.8 | 1.863929 | 97.1524 |
| 221.0935 | 2.44955 | 0.301483 | 2 | 100 - 1.4 | HMDB28848 | Glycyl-Phenylalanine | C11H14N2O3 | 41.7 | 22.1 | 1.694243 | 88.64518 |
| 579.1365 | 5.89145 | 0.058633 | 7 | 100 - 5.22 | HMDB38469 | Graveobioside A | C26H28O15 | 44.3 | 46.9 | 1.624454 | 76.46863 |
| 285.0772 | 7.893017 | 0.134 | 21 | 100 - 14.9 | HMDB31420 | Heliannone C | C16H14O5 | 47.9 | 45.7 | 1.268886 | 95.39506 |
| 201.1136 | 7.8009 | 0.150733 | 7 | 100 - 8.09 | HMDB59719 | Heptylmalonic acid | C10H18O4 | 45.4 | 32.9 | 1.629062 | 95.9347 |
| 413.1637 | 10.10387 | 0.167483 | 3 | 100 - 16.3 - 5.85 | HMDB41256 | Heteroflavanone C | C23H26O7 | 42.5 | 29.4 | 7.636229 | 91.62748 |
| 285.2074 | 9.425533 | 0.117233 | 4 | 100 - 7.53 | 5642 | Hexadecanedioic acid | C16H30O4 | 41.4 | 18.8 | 0.868092 | 89.35939 |
| 195.0664 | 6.703767 | 0.24285 | 18 | 100 - 9.87 - 0.207 | 24093 | Homoveratric acid | C10H12O4 | 50.7 | 56.2 | 0.543771 | 97.76259 |
| 181.0508 | 3.621967 | 0.41035 | 21 | 100 - 1.09 | 34515 | Hydroxyphenyllactic acid | C9H10O4 | 48.7 | 53.9 | 1.187622 | 90.84597 |
| 258.1465 | 1.82985 | 0.226117 | 10 | 100 - 1.75 | HMDB28868 | Hydroxyprolyl-Lysine | C11H21N3O4 | 46 | 44.4 | 2.273024 | 88.33916 |
| 173.0721 | 6.486033 | 0.092133 | 4 | 100 - 3.3 | 3302 | Indole-3-acetamide | C10H10N2O | 44.8 | 33 | 0.482989 | 91.80091 |
| 160.0404 | 7.189567 | 0.1005 | 7 | 100 - 0.83 | 3795 | Indole-3-carboxylic acid | C9H7NO2 | 44.7 | 33 | 0.096901 | 90.72335 |
| 174.0562 | 6.8545 | 0.11725 | 5 | 100 - 1.45 | HMDB00197 | Indoleacetic acid | C10H9NO2 | 46.2 | 41.3 | 0.656084 | 90.29074 |
| 204.0667 | 6.97175 | 0.184233 | 10 | 100 - 10.9 | 71 | Indolelactic acid | C11H11NO3 | 54.5 | 75.9 | 0.495503 | 97.29338 |
| 243.1718 | 3.479617 | 0.485717 | 4 | 100 - 5.32 | HMDB28910 | Isoleucyl-Isoleucine | C12H24N2O3 | 41.8 | 19.9 | 1.682411 | 90.98083 |
| 395.2055 | 8.17775 | 0.234483 | 1 | 100 - 21.6 | HMDB29622 | Isopetasoside | C21H32O7 | 44.8 | 34.6 | -5.05959 | 95.10168 |
| 187.0415 | 1.235267 | 0.318233 | 1 | 100 - 5.89 - 0.859 | HMDB35291 | Isoplumbagin | C11H8O3 | 41.4 | 21.2 | 7.431727 | 94.14307 |
| 315.0512 | 8.53785 | 0.092117 | 8 | 100 - 2.5 | 3445 | Isorhamnetin | C16H12O7 | 48.5 | 58.8 | 0.680062 | 84.7471 |
| 769.1989 | 6.586517 | 0.133983 | 3 | 100 - 38.3 | HMDB38252 | Isorhamnetin 3-O-[4-Hydroxy-E-cinnamoyl-(->6)-b-D-glucopyranosyl-(1->2)-a-L-rhamnopyranoside] | C37H38O18 | 45 | 37.3 | 0.520664 | 88.55997 |
| 609.1465 | 6.57815 | 0.092117 | 18 | 100 - 27.8 - 0.362 | HMDB40490 | Isorhamnetin 3-O-[b-D-xylopyranosyl-(1->6)-b-D-glucopyranoside] | C27H30O16 | 43.8 | 28.1 | 0.678406 | 91.91081 |
| 431.0987 | 6.502783 | 0.067 | 18 | 100 - 10.1 | 263495 | Isovitexin | C21H20O10 | 44.4 | 37 | 0.688523 | 85.56365 |
| 209.1183 | 10.49747 | 0.0335 | 11 | 100 | HMDB32797 | Jasmonic acid | C12H18O3 | 40.8 | 17.1 | 0.120807 | 87.07535 |
| 795.4546 | 9.023567 | 0.159117 | 4 | 100 - 49.2 - 8.26 | HMDB40946 | Jujubasaponin VI | C42H68O14 | 42.2 | 19.3 | 1.262616 | 93.25845 |
| 285.041 | 7.775783 | 0.234483 | 22 | 100 - 10.8 | HMDB05801 | Kaempferol | C15H10O6 | 45.4 | 36.5 | 1.804611 | 92.83451 |
| 212.0929 | 6.335283 | 0.142367 | 5 | 100 - 8.06 | 44252 | Kainic acid | C10H15NO4 | 44.7 | 28.3 | 0.130327 | 95.59231 |
| 89.02403 | 1.252017 | 0.401967 | 11 | 100 - 3.06 - 0.436 | 116 | Lactic acid | C3H6O3 | 44 | 25.8 | -4.2843 | 99.43575 |
| 131.046 | 1.084517 | 0.159117 | 7 | 100 - 2.22 - 0.126 | HMDB00168 | L-Asparagine | C4H8N2O3 | 47 | 40.7 | -1.99874 | 96.56053 |
| 132.0298 | 1.319 | 0.259617 | 7 | 100 - 2.67 - 0.203 | 15 | L-Aspartic Acid | C4H7NO4 | 52.9 | 70.7 | -2.86873 | 97.19723 |
| 245.1146 | 2.4663 | 0.1926 | 7 | 100 | HMDB11166 | L-beta-aspartyl-L-leucine | C10H18N2O5 | 41.8 | 22.7 | 1.431083 | 87.89757 |
| 317.1972 | 7.00525 | 0.108867 | 5 | 100 - 9.2 | HMDB29850 | L-Citronellol glucoside | C16H30O6 | 42.3 | 22 | 0.748755 | 90.31184 |
| 277.1559 | 5.866317 | 0.30985 | 4 | 100 - 16.2 - 0.146 | HMDB13243 | Leucyl-phenylalanine | C15H22N2O3 | 48.7 | 46.7 | 0.495328 | 97.31667 |
| 217.1188 | 1.101267 | 0.217733 | 9 | 100 - 7.66 | HMDB28938 | Leucyl-Serine | C9H18N2O4 | 45.6 | 35.2 | -2.66253 | 95.87948 |
| 231.1339 | 1.151517 | 0.175867 | 5 | 100 - 6.43 - 0.0682 | HMDB28939 | Leucyl-Threonine | C10H20N2O4 | 43.6 | 29.9 | -5.06302 | 93.81245 |
| 316.1669 | 6.033817 | 0.108867 | 4 | 100 - 4.09 | HMDB28940 | Leucyl-Tryptophan | C17H23N3O3 | 41.6 | 23.9 | 0.618118 | 84.91513 |
| 209.0661 | 1.168267 | 0.159117 | 8 | 100 - 6.22 | HMDB29901 | L-Galacto-2-heptulose | C7H14O7 | 44.6 | 29.9 | -2.96095 | 96.71123 |
| 245.1137 | 1.1264 | 0.192617 | 7 | 100 - 12.3 - 0.627 | HMDB11172 | L-gamma-glutamyl-L-valine | C10H18N2O5 | 47.1 | 39.8 | -2.48681 | 98.67923 |
| 146.0455 | 1.10965 | 0.418717 | 13 | 100 - 6.03 | 19 | L-Glutamate | C5H9NO4 | 54.8 | 78.1 | -2.64695 | 99.08673 |
| 145.0616 | 1.084517 | 0.167483 | 7 | 100 - 4.05 - 0.251 | HMDB00641 | L-Glutamine | C5H10N2O3 | 53.9 | 74.1 | -1.77751 | 97.33241 |
| 337.1088 | 9.475783 | 0.05025 | 12 | 100 - 2.09 | HMDB35373 | Licoagroaurone | C20H18O5 | 41.1 | 26.8 | 2.074373 | 81.18703 |
| 279.232 | 12.64132 | 0.30985 | 12 | 100 - 18.3 | HMDB00673 | Linoleic acid | C18H32O2 | 38.5 | 0 | -3.4929 | 96.68896 |
| 227.1405 | 2.4663 | 1.09705 | 4 | 100 - 8.06 | HMDB11174 | L-isoleucyl-L-proline | C11H20N2O3 | 41.2 | 13.9 | 1.791496 | 94.40054 |
| 201.1248 | 1.921967 | 0.217733 | 6 | 100 - 3.44 | 23819 | L-Leucyl-L- Alanine | C9H18N2O3 | 46.9 | 44.2 | 1.617398 | 92.41593 |
| 130.0874 | 1.687483 | 0.368483 | 13 | 100 - 1.04 | HMDB01645 | L-Norleucine | C6H13NO2 | 43.2 | 22.6 | 0.134983 | 93.81485 |
| 164.0719 | 1.930333 | 0.527583 | 12 | 100 - 8.14 | 28 | L-Phenylalanine | C9H11NO2 | 55 | 79.6 | 1.471716 | 97.25865 |
| 261.1247 | 5.46435 | 0.66995 | 4 | 100 - 7.61 | 23997 | L-phenylalanyl-L-proline | C14H18N2O3 | 41.8 | 19 | 0.957879 | 91.04353 |
| 173.0927 | 1.134767 | 0.167483 | 7 | 100 - 11.8 - 1.32 | HMDB34365 | L-Theanine | C7H14N2O3 | 42.9 | 20.5 | -2.56973 | 96.81726 |
| 203.0829 | 2.91015 | 0.963067 | 11 | 100 - 10.6 | 33 | L-Tryptophan | C11H12N2O2 | 50.4 | 56.7 | 1.633687 | 97.00821 |
| 502.253 | 5.7407 | 0.167483 | 4 | 100 - 20.1 - 2.06 | 168462 | Lys Asp Leu Glu | C21H37N5O9 | 43.6 | 27.5 | 2.24193 | 93.2598 |
| 375.214 | 5.631833 | 0.159117 | 16 | 100 - 7.05 | HMDB30455 | Macrophorin C | C22H32O5 | 42.9 | 41.8 | -9.70093 | 83.19354 |
| 103.0034 | 1.653983 | 0.3936 | 5 | 100 - 1.88 - 0.276 | 3237 | Malonic acid | C3H4O4 | 48.6 | 48.5 | -2.72529 | 97.93991 |
| 330.2036 | 5.832817 | 0.242867 | 1 | 100 - 10.5 | HMDB14924 | Marimastat | C15H29N3O5 | 41 | 13.9 | 0.450882 | 91.5933 |
| 431.1746 | 8.680217 | 0.318233 | 5 | 100 - 14.9 - 0.366 | HMDB35865 | Melledonal A | C23H28O8 | 41.9 | 30.5 | 7.89383 | 88.00397 |
| 399.1828 | 11.66152 | 0.066983 | 2 | 100 - 16.9 | HMDB35689 | Melleolide | C23H28O6 | 46.3 | 45.9 | 3.635041 | 89.68342 |
| 253.1083 | 6.51115 | 0.4271 | 1 | 100 - 12.4 | HMDB32796 | Methyl 3-(2,3-dihydroxy-3-methylbutyl)-4-hydroxybenzoate | C13H18O5 | 40.3 | 5.92 | 0.575661 | 96.39751 |
| 205.0345 | 1.344133 | 0.150733 | 9 | 100 | HMDB06471 | Methylisocitric acid | C7H10O7 | 42.7 | 27 | -4.12019 | 91.08658 |
| 315.2543 | 9.802383 | 0.142367 | 4 | 100 - 13 | HMDB11532 | MG(0:0/15:0/0:0) | C18H36O4 | 40.1 | 9.44 | 0.727524 | 91.81117 |
| 381.2651 | 11.08367 | 0.092117 | 8 | 100 - 10.7 | HMDB15064 | Misoprostol | C22H38O5 | 41.2 | 21.6 | 1.158908 | 85.95903 |
| 236.0773 | 1.319 | 0.1005 | 12 | 100 - 3.77 | HMDB37848 | N-(1-Deoxy-1-fructosyl)glycine | C8H15NO7 | 45.4 | 35.2 | -1.20131 | 93.11813 |
| 187.1083 | 1.1264 | 0.1926 | 10 | 100 - 7.18 - 0.101 | HMDB00206 | N6-Acetyl-L-lysine | C8H16N2O3 | 46.9 | 40.9 | -2.59063 | 96.89234 |
| 190.0544 | 3.915083 | 0.602967 | 2 | 100 | 62466 | N-Acetyl-DL-methionine | C7H13NO3S | 45.6 | 41.8 | 0.541532 | 87.00886 |
| 188.056 | 1.218517 | 0.334967 | 5 | 100 - 54.3 - 1.16 | 3325 | N-Acetyl-L-glutamic acid | C7H11NO5 | 44 | 52.8 | -2.51358 | 70.32204 |
| 206.0824 | 6.712133 | 0.142367 | 7 | 100 - 1.31 | HMDB00512 | N-Acetyl-L-phenylalanine | C11H13NO3 | 44.4 | 33.6 | 0.406958 | 88.89519 |
| 187.1091 | 1.913583 | 0.24285 | 10 | 100 - 1.42 | HMDB00446 | N-Alpha-acetyllysine | C8H16N2O3 | 43.2 | 26.5 | 1.721426 | 91.60164 |
| 347.1706 | 11.52752 | 0.108867 | 10 | 100 - 19.5 | HMDB39014 | Nepetariaside | C16H28O8 | 43.9 | 25.7 | -1.5719 | 95.85031 |
| 235.0728 | 1.972217 | 0.167483 | 2 | 100 | HMDB01200 | N'-Formylkynurenine | C11H12N2O4 | 44.5 | 37.4 | 1.772323 | 87.22743 |
| 202.1086 | 6.5279 | 0.3266 | 2 | 100 - 9.23 - 0.214 | HMDB62176 | N-lactoyl-Leucine | C9H17NO4 | 47.4 | 39.7 | 0.45928 | 97.92642 |
| 236.0929 | 6.821 | 0.217733 | 4 | 100 - 14.1 - 0.346 | HMDB62175 | N-lactoyl-Phenylalanine | C12H15NO4 | 47 | 37.3 | 0.453871 | 98.33688 |
| 188.0929 | 5.079133 | 0.1675 | 4 | 100 - 0.773 | HMDB62181 | N-lactoyl-Valine | C8H15NO4 | 41.1 | 14.9 | 0.443228 | 91.15295 |
| 269.0456 | 9.375283 | 0.15075 | 19 | 100 - 1.58 | HMDB29521 | Norizalpinin | C15H10O5 | 41.2 | 20.8 | 0.070423 | 85.31609 |
| 264.088 | 6.6619 | 0.1005 | 3 | 100 - 3.47 | HMDB59772 | N-Phenylacetylglutamic acid | C13H15NO5 | 43.2 | 28.8 | 0.884222 | 88.41603 |
| 218.1038 | 2.265317 | 0.267983 | 5 | 100 - 1.16 | 241 | Pantothenic Acid | C9H17NO5 | 46.2 | 43 | 1.843733 | 90.21803 |
| 193.0349 | 1.1599 | 0.167483 | 14 | 100 - 5.65 - 7.12 | HMDB03363 | Pectic acid | C6H10O7 | 45.4 | 36 | -2.62863 | 94.04188 |
| 269.0458 | 7.273317 | 0.3601 | 19 | 100 - 13.6 | 3399 | Pelargonidin | C15H10O5 | 50.6 | 58.6 | 1.020667 | 95.42562 |
| 377.1949 | 9.735383 | 0.1005 | 9 | 100 - 18.6 | HMDB03933 | Pentosidine | C17H26N6O4 | 43 | 21.5 | 1.691751 | 95.43199 |
| 296.1145 | 2.18995 | 0.527583 | 1 | 100 - 5.24 | HMDB10323 | Phenethylamine glucuronide | C14H19NO6 | 42 | 23.7 | 1.952275 | 88.71118 |
| 165.0559 | 6.728883 | 0.318233 | 34 | 100 - 9.7 | 34529 | Phenyllactic acid | C9H10O3 | 56.2 | 83.2 | 0.832391 | 98.80804 |
| 273.0762 | 8.2615 | 0.175867 | 25 | 100 - 13.6 | HMDB03306 | Phloretin | C15H14O5 | 46.8 | 41.4 | -2.41836 | 95.37327 |
| 435.13 | 5.9082 | 0.150733 | 5 | 100 - 12.5 - 0.106 | HMDB36634 | Phlorizin | C21H24O10 | 41.4 | 20.4 | 0.694978 | 87.49042 |
| 281.1397 | 6.08405 | 0.167483 | 9 | 100 - 12.8 - 0.37 | HMDB39241 | Pisumic acid | C15H22O5 | 45.3 | 32.7 | 0.814456 | 94.90639 |
| 939.4969 | 9.29155 | 0.234483 | 16 | 100 - 57.6 - 12 | HMDB38317 | Pisumsaponin II | C48H76O18 | 43.4 | 26.7 | 1.04018 | 91.54456 |
| 243.1243 | 7.541283 | 0.125617 | 1 | 100 - 8.11 | HMDB32472 | Polyethylene, oxidized | C12H20O5 | 48.1 | 49.2 | 1.975105 | 93.66809 |
| 225.0885 | 2.064333 | 0.418717 | 3 | 100 - 7.95 | HMDB00245 | Porphobilinogen | C10H14N2O4 | 46.5 | 39.4 | 1.682903 | 95.15926 |
| 259.0613 | 6.946617 | 0.100483 | 4 | 100 - 0.517 | HMDB40630 | Pratenol A | C14H12O5 | 41.5 | 22.8 | 0.548462 | 85.3133 |
| 227.1042 | 3.13625 | 0.418717 | 4 | 100 - 7.18 | HMDB06695 | Prolylhydroxyproline | C10H16N2O4 | 40.5 | 10.6 | 1.984596 | 94.45763 |
| 73.0294 | 1.69585 | 0.2931 | 8 | 100 - 0.906 | 3215 | Propionic acid | C3H6O2 | 43.9 | 24.1 | -1.33578 | 97.17404 |
| 339.2543 | 10.05362 | 0.0335 | 4 | 100 - 12.8 | 45415 | Prostaglandin D1 Alcohol | C20H36O4 | 40.8 | 15.1 | 0.726255 | 89.74016 |
| 137.0244 | 5.355483 | 0.452217 | 13 | 100 - 2.2 | 3263 | p-Salicylic acid | C7H6O3 | 54.9 | 80.7 | 0.067221 | 94.08377 |
| 184.098 | 6.51115 | 0.150733 | 2 | 100 - 1.9 | HMDB06348 | Pseudoecgonine | C9H15NO3 | 43.7 | 27.8 | 0.533705 | 91.40108 |
| 271.0706 | 2.901767 | 0.318233 | 2 | 100 - 7.23 | HMDB61177 | Pyridine N-oxide glucuronide | C11H14NO7+ | 41.6 | 18.3 | 3.163072 | 93.1782 |
| 128.0349 | 1.218517 | 0.175867 | 9 | 100 - 4.92 - 0.218 | HMDB00267 | Pyroglutamic acid | C5H7NO3 | 46.6 | 38.1 | -3.16319 | 98.50303 |
| 301.0358 | 7.39055 | 0.117233 | 11 | 100 - 8.15 | HMDB05794 | Quercetin | C15H10O7 | 46 | 41.5 | 1.566882 | 90.39945 |
| 297.2431 | 11.24278 | 0.3601 | 10 | 100 - 19.5 - 0.65 | HMDB34297 | Ricinoleic acid | C18H34O3 | 43.5 | 21.3 | -1.4301 | 97.89697 |
| 431.1726 | 9.5344 | 0.075367 | 3 | 100 - 11.8 | 85003 | SALVINORIN A | C23H28O8 | 43.6 | 36.4 | 3.293483 | 85.35656 |
| 217.1198 | 1.79635 | 0.15075 | 9 | 100 - 0.738 | HMDB29042 | Serinyl-Isoleucine | C9H18N2O4 | 44.2 | 33.4 | 1.808235 | 89.71883 |
| 251.1043 | 2.2737 | 0.251217 | 6 | 100 | HMDB29046 | Serinyl-Phenylalanine | C12H16N2O4 | 43.4 | 33.3 | 2.116492 | 86.25554 |
| 223.0616 | 6.862883 | 0.058633 | 6 | 100 - 3.56 | 45738 | Sinapic acid | C11H12O5 | 48 | 51.4 | 1.716902 | 90.77875 |
| 345.2289 | 7.516167 | 0.267983 | 1 | 100 - 11.4 | HMDB29885 | Sorbitan laurate | C18H34O6 | 40.7 | 15.6 | 1.766895 | 90.16923 |
| 957.5077 | 8.646717 | 0.142367 | 10 | 100 - 53.9 - 6.09 | HMDB38608 | Soyasaponin A3 | C48H78O19 | 42.4 | 23.4 | 1.296419 | 89.94837 |
| 941.5127 | 8.76395 | 0.24285 | 14 | 100 - 53.5 - 13.4 - 0.389 | HMDB34649 | Soyasaponin I | C48H78O18 | 46.6 | 39.8 | 1.199197 | 94.67304 |
| 911.5019 | 8.93145 | 0.184233 | 3 | 100 - 51.6 - 13.2 - 0.255 | HMDB34650 | Soyasaponin II | C47H76O17 | 45.5 | 34.3 | 1.040368 | 94.69235 |
| 309.2072 | 9.425533 | 0.27635 | 8 | 100 - 8.72 | HMDB35337 | Sterebin A | C18H30O4 | 47.2 | 47.8 | 0.292131 | 88.42119 |
| 173.082 | 6.6284 | 0.142367 | 7 | 100 - 5.61 | 4243 | Suberic acid | C8H14O4 | 49 | 49.8 | 0.396084 | 95.88629 |
| 230.1035 | 6.335283 | 0.150733 | 3 | 100 - 3.33 | HMDB00953 | Suberylglycine | C10H17NO5 | 44 | 29.7 | 0.582788 | 91.16859 |
| 117.0189 | 1.285517 | 0.3601 | 8 | 100 - 4.09 - 0.0993 | 114 | Succinic acid | C4H6O4 | 51.8 | 64.1 | -3.42765 | 98.80853 |
| 157.0507 | 5.749083 | 0.284733 | 5 | 100 - 1.58 | 4154 | Succinylacetone | C7H10O4 | 44.1 | 27.7 | 0.32638 | 93.2522 |
| 461.1094 | 6.586517 | 0.092133 | 11 | 100 - 14 | HMDB30569 | Swertiajaponin | C22H22O11 | 43.8 | 32.7 | 1.083883 | 87.48615 |
| 197.0456 | 6.042183 | 0.1675 | 9 | 100 - 1.32 | 2256 | Syringic acid | C9H10O5 | 49.4 | 56.8 | 0.479353 | 90.7646 |
| 229.1447 | 7.047117 | 0.150817 | 2 | 100 - 10.8 | HMDB30143 | Talaromycin A | C12H22O4 | 45.7 | 33 | 0.647027 | 96.22848 |
| 293.1757 | 9.693517 | 0.125617 | 7 | 100 - 3.63 | HMDB35722 | Tanacetol A | C17H26O4 | 44.1 | 35.8 | -0.42552 | 85.23599 |
| 257.176 | 8.57135 | 0.134 | 5 | 100 - 2.21 | HMDB00872 | Tetradecanedioic acid | C14H26O4 | 42.8 | 27.8 | 0.615441 | 86.82074 |
| 474.2578 | 3.37075 | 0.443833 | 4 | 100 - 21.1 | 238442 | Thr Val Lys Glu | C20H37N5O8 | 41.9 | 18.1 | 1.790464 | 93.7248 |
| 231.1354 | 1.905217 | 0.209367 | 5 | 100 - 1.91 | HMDB29065 | Threoninyl-Leucine | C10H20N2O4 | 41.8 | 21.1 | 1.735346 | 89.77312 |
| 265.1201 | 2.4998 | 0.267983 | 3 | 100 - 4.58 | HMDB29068 | Threoninyl-Phenylalanine | C13H18N2O4 | 42.5 | 26.1 | 2.533891 | 89.21897 |
| 271.0614 | 6.929867 | 0.142367 | 15 | 100 - 34.2 | HMDB38549 | Toralactone | C15H12O5 | 40.3 | 17.1 | 0.675297 | 85.22567 |
| 147.0452 | 6.728883 | 0.242867 | 9 | 100 - 5.95 | 63104 | trans-Cinnamic acid | C9H8O2 | 47.4 | 41.9 | 0.292501 | 95.66802 |
| 309.2073 | 10.47233 | 0.284733 | 8 | 100 - 19.4 | 35761 | trans-EKODE-(E)-Ib | C18H30O4 | 55.7 | 81.8 | 0.435695 | 97.08778 |
| 227.129 | 8.328483 | 0.133983 | 3 | 100 - 9.29 | 5882 | Traumatic Acid | C12H20O4 | 47.6 | 43.7 | 0.646612 | 94.91415 |
| 293.1151 | 1.7461 | 0.075367 | 8 | 100 - 8 | HMDB29106 | Tyrosyl-Hydroxyproline | C14H18N2O5 | 40.6 | 15 | 2.647901 | 90.93818 |
| 215.1291 | 7.3403 | 0.092133 | 2 | 100 - 25.6 | HMDB00888 | Undecanedioic acid | C11H20O4 | 40 | 13 | 1.084701 | 88.27257 |
| 431.215 | 4.38405 | 0.4606 | 2 | 100 - 17.4 | 243381 | Val Leu Ser Asp | C18H32N4O8 | 40.5 | 10.3 | 0.666418 | 93.22201 |
| 263.1403 | 4.25005 | 0.644833 | 6 | 100 - 7.16 | 24009 | Val Phe | C14H20N2O3 | 43.5 | 27.4 | 0.602212 | 90.63879 |
| 302.1513 | 5.573217 | 0.18425 | 3 | 100 - 8.03 | 23665 | Val Trp | C16H21N3O3 | 46.2 | 43.1 | 0.792955 | 89.09705 |
| 231.098 | 1.1264 | 0.200983 | 7 | 100 - 9.87 - 0.529 | HMDB29123 | Valyl-Aspartate | C9H16N2O5 | 47.7 | 43.8 | -2.68295 | 98.10845 |
| 229.1562 | 1.930333 | 0.20935 | 4 | 100 - 3.32 | HMDB29130 | Valyl-Isoleucine | C11H22N2O3 | 43.3 | 28.5 | 1.723702 | 90.23522 |
| 263.1406 | 3.22 | 0.1005 | 6 | 100 - 4.14 | HMDB29134 | Valyl-Phenylalanine | C14H20N2O3 | 40.3 | 15.6 | 1.814217 | 88.08561 |
| 151.0257 | 1.185017 | 0.251233 | 5 | 100 - 3.32 | 82 | Xanthine | C5H4N4O2 | 50.2 | 58.8 | -3.03765 | 96.01322 |
| 301.2025 | 7.742283 | 0.092117 | 3 | 100 - 9.49 | HMDB37830 | xi-7-Hydroxyhexadecanedioic acid | C16H30O5 | 41 | 16.3 | 1.659319 | 90.77109 |
| 193.087 | 9.25805 | 0.08375 | 22 | 100 - 3.47 | 71737 | Zingerone | C11H14O3 | 42.2 | 20 | 0.027598 | 91.18887 |
| 157.0137 | 1.218517 | 0.242867 | 3 | 100 - 3.84 | HMDB31210 | Zymonic acid | C6H6O5 | 44 | 27.5 | -3.29933 | 96.17806 |
